# Pretransplant Systemic Lipidomic Profiles in Allogeneic Stem Cell Transplant Recipients

**DOI:** 10.3390/cancers14122910

**Published:** 2022-06-13

**Authors:** Kimberley Joanne Hatfield, Øystein Bruserud, Håkon Reikvam

**Affiliations:** 1 Department of Immunology and Transfusion Medicine, Haukeland University Hospital, 5020 Bergen, Norway; hatf@helse-bergen.no; 2 Department of Clinical Science, University of Bergen, 5020 Bergen, Norway; hakon.reikvam@uib.no; 3 Department of Medicine, Haukeland University Hospital, 5021 Bergen, Norway

**Keywords:** graft-versus-host disease, hematopoietic stem cell transplantation, inflammation, leukemia, lipidomics, metabolic profiles, myelodysplastic syndromes

## Abstract

**Simple Summary:**

Stem cell transplantation is used in the treatment of aggressive hematological malignancies and consists of initial high-dose and potentially lethal chemotherapy, followed by rescue with the transplantation of hematopoietic stem cells. Transplantation with stem cells from a healthy donor (i.e., allogeneic stem cells) has the strongest anti-cancer effect, but also the highest risk of severe toxicity. Furthermore, the clinical status at the time of transplantation (inflammation, fluid overload) is associated with posttransplant mortality, and immune-mediated acute graft-versus-host disease (GVHD) is a potential lethal complication. Finally, lipid metabolism regulates the proliferation and survival of both malignant hematological cells and immunocompetent cells that cause GVHD. Our study shows that the pretransplant lipid profiles differ between allotransplant recipients and can be used for the subclassification of patients and possibly to identify patients with an increased risk of death due to disease relapse or treatment toxicity. The therapeutic targeting of lipid metabolism should therefore be further explored in these transplant recipients.

**Abstract:**

Allogeneic stem cell transplantation is used in the treatment of high-risk hematological malignancies. However, this treatment is associated with severe treatment-related morbidity and mortality. The metabolic status of the recipient may be associated with the risk of development of transplant-associated complications such as graft-versus-host disease (GVHD). To better understand the impact of the lipidomic profile of transplant recipients on posttransplant complications, we evaluated the lipid signatures of patients with hematological disease using non-targeted lipidomics. In the present study, we studied pretransplant serum samples derived from 92 consecutive patients with acute myeloid leukemia (AML) or high-risk myelodysplastic syndrome (MDS). A total of 960 lipid biochemicals were identified, and the pretransplant lipidomic profiles differed significantly when comparing patients with and without the risk factors: (i) pretransplant inflammation, (ii) early fluid overload, and (iii) patients with and without later steroid-requiring acute GVHD. All three factors, but especially patients with pretransplant inflammation, were associated with decreased levels of several lipid metabolites. Based on the overall concentrations of various lipid subclasses, we identified a patient subset characterized by low lipid levels, increased frequency of MDS patients, signs of inflammation, decreased body mass index, and an increased risk of early non-relapse mortality. Metabolic targeting has been proposed as a possible therapeutic strategy in allotransplant recipients, and our present results suggest that the clinical consequences of therapeutic intervention (e.g., nutritional support) will also differ between patients and depend on the metabolic context.

## 1. Introduction

Allogeneic stem cell transplantation is used in the treatment of various hematological malignancies. This treatment has the highest antileukemic efficacy; however, it is also the therapeutic alternative with the highest risk of severe toxicity and non-relapse mortality after transplantation. It consists of initial conditioning therapy that often includes total body irradiation and/or high-dose chemotherapy, and this treatment is followed by stem cell infusion. The conditioning can be either myeloablative or it can be a less toxic nonmyeloablative or reduced-intensity conditioning [1,2]. The myeloablative conditioning is generally associated with lower relapse risk, but is often regarded as the most toxic alternative, i.e., it has the highest risk of non-relapse mortality.

Due to the high risk of severe toxicity, allotransplantation is used mainly in the treatment of aggressive or high-risk malignancies [3,4]. The main causes of mortality after allotransplantation are the relapse of chemoresistant malignancy and non-relapse mortality, including infections, immune-mediated graft-versus-host disease and transplant-related toxicity. The risk of non-relapse mortality is caused by various factors. First, an increased risk of severe toxicity is seen for patients with other diseases. The comorbidity index is used in the pretransplant evaluation of these patients, and it reflects the impact of various concomitant diseases on the risk of posttransplant toxicity [5,6]. Second, the impact of immunological factors (including aging) is reflected in the European Group for Blood and Marrow Transplantation (EBMT) index, i.e., patient age, type of donor, human leukocyte antigen (HLA) match/mismatch, and donor/recipient sex combination [7]. Third, certain factors reflecting the biological status of the patients at the time of transplantation also seem to have a prognostic impact. The most important factors are systemic signs of inflammation reflected in the pretransplant level of C-reactive protein (CRP), signs of endothelial dysfunction reflected either in the Endothelial Activation and Stress Index (EASIX) that is defined as lactate dehydrogenase (U/L) × creatinine (mg/dL)/platelets (10^9^ cells/L) or in fluid overload/retention early after conditioning and/or stem cell infusion [8]. The impact of inflammation/endothelial dysfunction may also at least partially be reflected in the EBMT index that includes disease stage and time from diagnosis to transplant [7].

Severe acute or chronic graft-versus-host disease (GVHD), systemic signs of inflammation and endothelial dysfunction with fluid overload are relatively common posttransplant conditions that are associated with an adverse prognosis and decreased survival due to increased non-relapse mortality in allotransplant recipients. The impact of GVHD has been documented in several studies [9], and the association between increased mortality and inflammation has been documented both in single institution studies [10,11,12], and recently also in a meta-analysis [13]. Finally, the prognostic impact of early fluid overload complications has been documented in several recent studies, including the first study by Tvedt et al. in 2015 that investigated patients transplanted with stem cells derived from matched family donors [12]; the prognostic impact has later been described for matched unrelated donor transplants and haploidentical grafts [14], patients allotransplanted with CD34^+^ enriched cells, patients receiving GVHD prophylaxis without calcineurin inhibitors [15], allotransplanted children [16], in allotransplanted children with respiratory failure [17] and with engraftment syndrome [18], and after cord blood transplantation [19].

We have previously shown that there is an association between systemic metabolic profiles and the risk of later GVHD, as well as fluid retention after allotransplantation [20,21,22,23]. In the present study, we have further analyzed the pretransplant serum lipidomic profiles (i.e., a large number of molecular lipid species) for a consecutive group of allotransplant recipients diagnosed with AML or high-risk MDS. Our study focuses on investigating potential associations between lipidomic profiles and pretransplant inflammation, early fluid overload and later development of acute GVHD.

## 2. Materials and Methods

### 2.1. Patients

All patients were included after written informed consent and in accordance with the Declaration of Helsinki. The regional ethics committee approved the inclusion of samples into a registered biobank (REK Vest 1759/2015) and the use of samples in the present study (REK Vest 305/2017). Our department is responsible for allogeneic stem cell transplantation with family donors in a defined geographical area of Norway, and the present patients represent a consecutive group (Table 1). Our study should therefore be regarded as population based. The median follow-up was 104 months, and all patients had a follow-up time of at least 25 months.

None of the patients received parenteral nutrition or supplementary nutrition by an enteral tube; they were allowed to have their ordinary diet.

### 2.2. Analysis of Metabolic Profiles

Pretransplant samples were collected from all patients before the start of the pretransplant conditioning therapy. Venous blood was collected into sterile plastic tubes (BD Vacutainer^®^ SST™ Serum Separation Tubes, Becton-Dickenson; Franklin Lakes, NJ, USA) and allowed to coagulate for 120 min at room temperature before centrifugation (300× *g* for 10 min) according to a standard protocol. The serum was immediately aliquoted and stored at −80 °C until analyzed. All samples were analyzed by Metabolon (Morrisville, NC, USA) using the Metabolon^®^ Complex Lipid Platform (Supplementary material) [25,26].

### 2.3. Statistical and Bioinformatical Analyses

The Welch’s two-sample *t*-test was used to test whether two unknown means were different from two independent populations. For statistical significance testing, *p* values are given for individual biochemicals (Welch’s two-sample *t*-test). The False Discovery Rate (FDR) for a given set of compounds was estimated using the *q* value [27]. The overall data were analyzed using principal component analysis and the random forest classification technique.

Hierarchical cluster analyses were performed using the J-Express software (MolMine AS, Bergen, Norway). For standardization of the data before hierarchical clustering, z-transformation of the data was performed. In short, this was done by subtracting the average value from the observed values and then dividing it by the standard deviation; hence, taking into account the different scales and ranges of the metabolites. All values were median variance standardized and log(2) transformed; the complete linkage was used as the linkage method and for distance measured the Pearson correlation was used. Additional statistical analyses were performed using the Statistical Package for the Social Sciences (SPSS) (SPSS Inc., Chicago, IL, USA).

## 3. Results

### 3.1. Patients Included in the Study

We investigated 93 consecutive allotransplant patients diagnosed with either AML (de novo and AML secondary to MDS) or high-risk MDS. All patients were transplanted with granulocyte colony-stimulating factor (G-CSF) mobilized peripheral blood stem cell grafts derived from HLA-matched family donors. The characteristics of the patients are summarized in Table 1. All patients had a comorbidity score ≤ 3 [28].

### 3.2. The Frequencies of Patients with Systemic Signs of Inflammation and Excessive Postconditioning Fluid Retention

We investigated the associations between systemic signs of inflammation and excessive postconditioning fluid retention for all allotransplanted patients (Table 2). Both factors were present for 21 patients; both factors were absent in 38 patients; only fluid retention was detected for 16 patients and only inflammation for 14 patients (missing information on fluid retention for four transplantation patients). Thus, only one of the two factors was present for 30 patients, but despite this patient heterogeneity, we observed a significant association between inflammation and fluid retention when comparing the overall results (Fisher’s exact test, *p* = 0.0078).

### 3.3. The Frequency of Lipid Metabolites between Patients with versus without Fluid Overload/Inflammation/Acute GVHD

The number of biochemicals/metabolites that differed significantly between patients with and without fluid overload, inflammation or acute GVHD are presented in Table 2. A total of 960 biochemicals belonging to 14 different lipid classes, were analyzed in the Complex lipid platform, and with a statistical significance corresponding to *p* < 0.05, we would then expect to detect by coincidence alone 48 biochemicals. Furthermore, if the differentially expressed biochemicals were detected by coincidence alone we would also expect a similar number of metabolites with significantly increased and decreased levels for each of the three patient comparisons. Acute GVHD patients, and especially patients with pretransplant signs of inflammation, showed a significantly higher number of differing biochemicals compared with the number that would be expected by coincidence alone, and all three comparisons showed that the differentially expressed biochemicals for patients with fluid overload/inflammation/acute GVHD differed significantly from the expected equal distribution between increased and decreased biochemicals.

### 3.4. The Pretransplant Total Levels of Various Lipid Species Differ Significantly between Patients with and without Pretransplant Inflammation, Early Fluid Overload and/or Later Acute GVHD

We investigated the total pretransplant serum levels of the 14 lipid classes/subclasses, namely phosphatidylcholines (PC), lysophosphatidylcholines (LPC), phosphatidylethanolamines (PE), lysophosphatidylethanolamines (LPE), phosphatidylinositols (PI), ceramides (Cer), dihydroceramides (DhCer), hexosylceramides (HexCer), lactosylceramides (LCER), sphingomyelins (SM), cholesteryl esters (CE), diacyglycerols (DAG), triacylglycerols (TAG) and monoacylglycerols (MAG). Only seven of these lipid subclasses showed significant differences between patients with pretransplant inflammation or posttransplant acute GVHD, and the results for these seven classes are summarized in Table 3 and Table S1. First, patients with systemic pretransplant signs of inflammation showed significantly decreased levels of total lysophophatidylcholine, lysophosphatidylethanolamine, phosphatidylinositol and cholesteryl esters. The most significant difference was observed for lysophosphatidylcholine (*p* = 0.0001). In contrast, excessive fluid overload was only associated with significantly decreased pretransplant levels of phosphatidylinositols, and this difference reached only borderline significance (*p* = 0.0499, *r* = 0.6958). Finally, acute GVHD was associated with significant differences for three other subclasses, namely sphingolipid/lactosylceramide, diacylglycerols and monoacylglycerols. For all three comparisons, the levels of all or almost all significantly altered metabolites were decreased in these patients.

To conclude, significantly decreased total serum levels were observed especially for patients with pretransplant inflammation or posttransplant acute GVHD, but these two patient subsets showed significant differences for different lipid subclasses and the association between inflammation and decreased total lysophosphatidylcholines was particularly strong (Table 3).

### 3.5. Hierarchical Cluster Analysis Based on the the Overall Pretransplant Concentrations of Various Lipid Subclasses Identifies Distinct Patient Subsets

**Figure 1:**
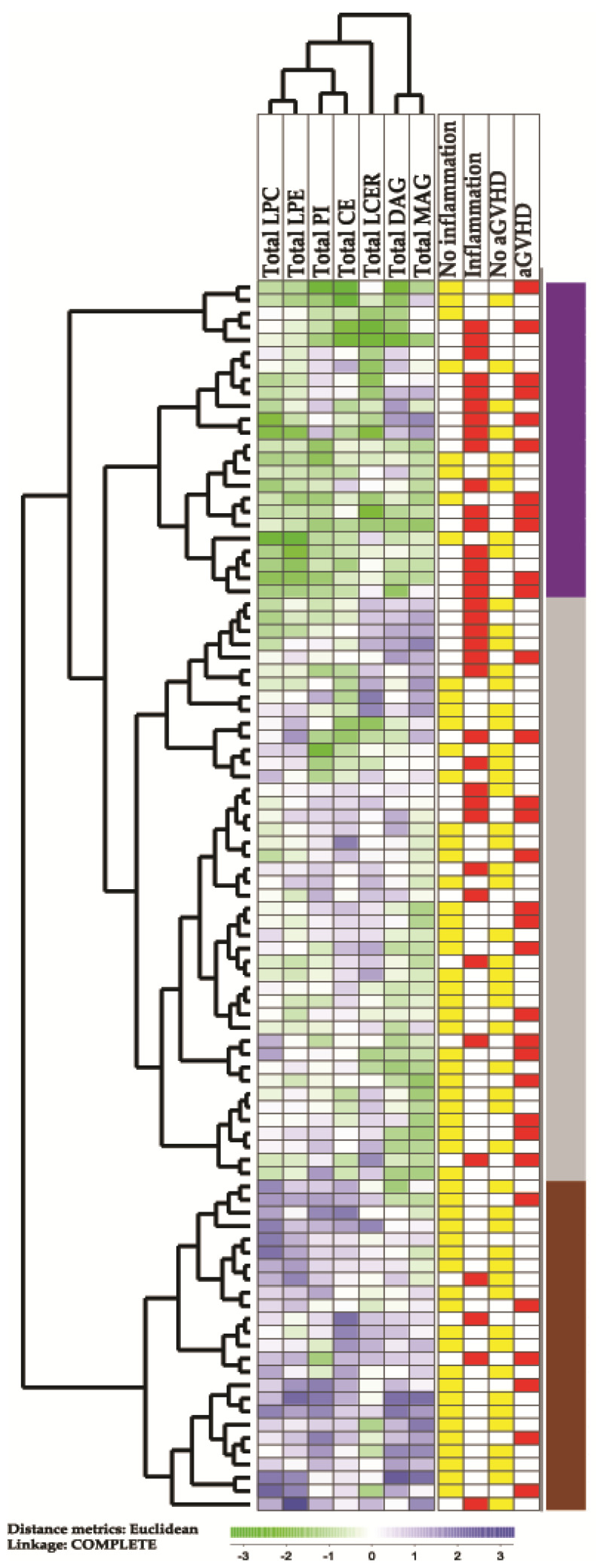
An unsupervised hierarchical cluster analysis based on the total concentrations of lipid metabolites in pretransplant serum samples derived from 93 allogeneic stem cell transplant recipients. In this analysis, we included the total serum concentrations of lysophophatidylcholine (LPC), lysophosphatodylethanolamine (LPE), phosphatidylinositol (PI), cholesteryl esters (CE), sphingolipid/lactosylceramide (LCER), diacylglycerols (DAG) and monoacylglycerols (MAG); the lipid levels were significantly altered when comparing recipients with and without signs of inflammation and/or patients with and without later acute GVHD grade 2–4. Green indicates low levels, while purple indicates high levels of metabolites for individual patients. The column to the far right indicates the three patient subsets identified by clustering, showing varied levels of lipids.

We did an unsupervised hierarchical cluster analysis based on the total pretransplant serum concentrations of the seven subclasses lysophophatidylcholine (LPC), lysophosphatodylethanolamine (LPE), phosphatidylinositol (PI), cholesteryl esters (CE), sphingolipid/lactosylceramide (LCER), diacylglycerols (DAG) and monoacylglycerols (MAG); these lipid subclasses showed statistically significant differences when comparing patients with pretransplant inflammation and/or later acute GVHD. The results from this analysis are presented in Figure 1. Three main patient subsets were identified: (i) an upper subset including 24 patients showing generally low levels, (ii) an intermediate subset including 44 patients and (iii) a lower subset including 25 patients with generally high levels of the various lipid subclasses.

We compared the contrasting upper main subset of 24 patients showing generally low levels of lipids (Figure 1) with the 25 patients in the lower subset showing generally high lipid levels, and a comparison of the clinical parameters for these subsets are shown in Table 4. The upper subset of Figure 1 included a group of patients with higher age (median age 56 versus 48 years, Mann–Whitney U test, *p* = 0.031) and increased frequencies of patients showing increased CRP levels (16 versus 4 patients, Fisher’s exact test, *p* = 0.0004) and preleukemic MDS (12 versus 3 patients, *p* = 0.0054). The upper subset also showed an increased frequency of early (i.e., before four months) non-relapse mortality, but this difference reached only borderline significance (six versus 1 patient, *p* = 0.042) and it should also be emphasized that the patients differed with regard to conditioning therapy. Furthermore, the pretransplant BMI was also slightly lower for patients in the upper subset (median 23.7 versus 24.6, *p* = 0.04036), but it should be emphasized that the median BMI was within the suggested normal range (18.5–25 kg/m^2^) [29] for both patient subsets/clusters. The two patient subsets did not differ with regard to time to posttransplant hematological reconstitution, excessive fluid overload or later acute GVHD. The frequencies of patients in pretransplant first versus second complete remission did not differ between the two contrasting clusters either, and the frequencies of patients with de novo AML and MDS-AML did not differ significantly between the clusters (data not shown). Finally, the difference could not be explained by longer storage time and degradation of metabolites for the upper subset that included patients with generally low lipid levels.

We also compared the 24 patients in the upper main cluster (Figure 1) with all the other 69 patients in the lower main cluster. These comparisons also demonstrated the increased frequencies of patients with previous MDS (*p* = 0.0388) and pretransplant signs of inflammation (*p* = 0.0016) and decreased BMI (*p* = 0.0394). As in the previous analysis, the increased frequency of patients with early non-relapse death reached only borderline significance (*p* = 0.0719); acute GVHD did not differ between the groups and there was no longer any significant difference in age (data not shown).

We then further investigated the associations between signs of inflammation (i.e., increased CRP levels) and MDS/MDS-AML, but we could not detect any significant difference in CRP levels between MDS/MDS-AML patients and the other patients (data not shown). Furthermore, myeloablative busulfan-cyclophosphamide conditioning was used especially for patients regarded to have high-risk disease, including eight of the 11 patients with MDS-AML.

We finally compared the two contrasting patient subsets identified in the cluster analysis (Figure 1) with regard to the levels of the seven lipid classes that were included in this analysis (Table 5). The most significant differences between these two contrasting patient subsets were observed for LPC esters and cholesteryl esters, but highly significant *p* values were also detected for LPE esters and monoacylglycerol esters. Thus, these four lipid classes/subclasses seem to be most important for the identification of these two contrasting patient subsets, i.e., for the identification of the upper main subset characterized by generally low total levels of metabolites belonging to these lipid classes, signs of inflammation and MDS/MDS-AML.

### 3.6. Analysis of Patient Heterogeneity Based on Principal 2-Component Analyses; Studies of Patients with and without Inflammation, Fluid Overload or Acute GVHD

As an initial analysis of the overall biochemical, we did principal 2-component analyses, but this analytical approach could not be used to distinguish between patients with and without inflammation/fluid overload/acute GVHD (data not shown).

### 3.7. Associations between the Serum Levels of Individual Lipid Metabolites and Pretransplant Inflammation, Early Fluid Overload and Posttransplant Acute GVHD

We first compared the pretransplant levels of all individual lipid metabolites for patients with and without pretransplant inflammation (Table S2). A total of 117 metabolites differed significantly between these two patient subsets, and all except one metabolite showed decreased levels in patients with pretransplant inflammation (Table 2). A classification of the differentially expressed metabolites is presented in Table 6, and a simplified visualization of these results is presented in Figure S1. The four largest subsets were phosphatidylcholine esters, lysophosphatidylcholine esters, plasmalogens and cholesteryl esters, which comprised 76 of the 117 metabolites. We also ranked the 117 metabolites based on their *p* values (Table S2); the lysophosphatidylcholine esters then constituted 11 of the 15 top-ranked metabolites and 10 out of the 13 metabolites with a *p* value <0.001. These observations are also consistent with our previous analyses of total lipid metabolite levels (Table 2).

Only 32 lipid metabolites differed significantly between patients with and without early fluid overload, which were spread between several lipid subclasses (Table S3), and only a diverse minority of six lipid metabolites showed a *p* value < 0.01 (Table S3). However, these 32 significantly altered metabolites showed decreased levels in the patients with fluid overload, and fifteen of the 32 biochemicals that showed significant differences when comparing the patients with and without fluid overload (Table 2, Welch *t*-test, *p* < 0.05) could also be detected when comparing patients with and without signs of inflammation (Table S4, upper part). Phosphatidylinositol (PI) esters constituted the relatively largest group (six out of 23 biochemicals analysed) and three of these metabolites were among the ten top-ranked lipid metabolites (i.e., with the highest significance, see Table S3); these observations are consistent with our previous analyses of total levels of lipid subclasses (see Section 3.4).

However, there was only a minor overlap between both acute GVHD and fluid overload/retention (Table S4, middle part), and also between acute GVHD and inflammation (Table S4, lower part) (six biochemicals for each of these two overlapping groups).

A total of 69 pretransplant lipid metabolites differed significantly when comparing patients with and without later acute GVHD requiring systemic steroid treatment (Table 6 and Table S5). Mono-, di- and triacylglycerols comprised 57 of these 69 metabolites, and 12 of the 15 top-ranked (i.e., most significant) metabolites were acylglycerols (Table S5). These observations are also consistent with our previous analyses of total levels of lipid subclasses (see Section 3.4). Furthermore, significantly altered lipid metabolites for patients with and without later acute GVHD showed only a minor overlap with the altered metabolites for patients with and without inflammation (Table S4, lower part, six metabolites) and fluid overload (Table S4, middle part, six metabolites).

Finally, only the two phosphatidylcholines PC(16:0/20:1) and PC(18:2/22:5) showed significant differences for all three comparisons, i.e., fluid overload, inflammation and acute GVHD (Table S4).

### 3.8. Subclassification of Allotransplant Recipients Based on the Levels of Individual Lipid Metabolites

As an alternative to the hierarchical cluster analysis based on the total concentrations of various lipid subclasses (Figure 1), we did an unsupervised cluster analysis based on the total levels/concentrations of individual lipid biochemical (Figure S2). We included only the pretransplant levels of those biochemicals that differed significantly between patients with and without pretransplant risk factors (i.e., inflammation/excessive fluid overload) and/or patients with and without later development of steroid-requiring acute GVHD (Table 6). The overall cluster analysis is presented in the Supplementary material (Figure S2). The allotransplant recipients could then be divided into two main clusters/subsets that are referred to as the upper main cluster A including 39 patients and the lower main cluster B including 54 patients. Each of these main clusters could be further divided into the two subclusters, A1/A2 and B1/B2, respectively (Figure S2).

A major difference between the two main patient clusters was an increased frequency of patients with pretransplant signs of inflammation in the upper main cluster A (Figure S2; 22 out of 39 patients) compared with the lower main cluster B (14 out of 54 patients; Fisher’s exact test, *p* = 0.0047). The frequency of patients with pretransplant inflammation was particularly high in the upper A1 subcluster (12 out of 18 patients), and this difference was also significant when compared with the 75 other patients (Fisher’s test, *p* = 0.0135). The frequency of MDS patients was also significantly different when comparing the A1 subcluster (12 out of 18 patients) compared with the other 75 patients (16 out of 75; Fisher’s exact test, *p* = 0.0004). The A1 subcluster was also characterized by generally low lipid metabolite levels. The main clusters/subclusters did not differ between the development of excessive fluid overload or acute GVHD, early non-relapse mortality, or BMI (data not shown). The frequencies of patients in pretransplant first versus second complete remission did not differ between the main clusters/subclusters either; and the frequencies of patients with de novo AML or MDS-AML did not differ significantly between the clusters (data not shown).

By hierarchical clustering, the lipid biochemicals were also clustered into two main groups referred to as clusters 1 and 2, and each of these groups could be further divided into three subclusters 1.1/1.2/1.3 and 2.1/2.2/2.3, respectively (Figure S2). The distribution of the various lipid metabolites into the two main clusters is summarized in Table 7 and is presented in more detail for each of the six subclusters in Table S6. Lipid metabolites belonging to the same relatively large lipid subclass usually clustered close to each other, either in the same main cluster or among only a few of the subclusters; this was especially seen for the acylglycerols but also for the PC esters, LPC esters and PE esters/ethers/plasmalogen. Patients in the upper main patient cluster A (A1 and A2) had relatively low serum levels of lipid metabolites as shown in subclusters 1.1 (including mainly PC esters, PE esters/ethers/plasmalogens, CE esters) and subcluster 2.2 (including mainly PC esters, PE esters/ethers/plasmalogans). These lipid subclasses showed decreased levels especially in patients with pretransplant inflammation, and as expected patients in the upper cluster A showed an increased frequency of pretransplant systemic signs of inflammation.

## 4. Discussion

Various lipid classes and individual lipid metabolites are not only important for cellular metabolism, they are also important regulators of cellular proliferation and survival through their function as extracellular signaling molecules that bind to specific cellular receptors or modulate intracellular signaling through their molecular interactions in cellular membranes (e.g., anchoring of cell surface membrane molecules) [30,31,32,33,34,35,36,37,38,39,40,41,42]. Furthermore, pretransplant biological characteristics have an impact on prognosis/survival after allogeneic stem cell transplantation [13], and lipid metabolites also seem to be involved in the regulation of proliferation and survival of myeloid malignant cells [43,44]. In this context, we have investigated the pretransplant serum lipidomic profiles for a consecutive group of allotransplanted patients with AML or high-risk MDS. Our department is responsible for allogeneic stem cell transplantation with matched related donors in a defined geographical area, and we investigated a consecutive group of allotransplant recipients with AML or high-risk MDS. Our study should therefore be regarded as population based, and the patient heterogeneity is limited (i.e., the large majority of patients being in remission, receiving the same GVHD prophylaxis, mainly sibling donors). To the best of our knowledge, our present study is the first to characterize in detail the pretransplant lipidomic profiles for allotransplant adult recipients.

Various lipids are important for regulation of inflammation and immunity, and they also play a role in the regulation of survival and proliferation of myeloid malignant cells [42,45,46,47,48,49,50,51,52,53]. The aim of our present study was therefore to investigate the pretransplant systemic lipid profiles for patients receiving allogeneic stem cell transplantation for aggressive myeloid malignancies. We used a stepwise scientific approach in our study. First, using non-targeted lipidomics, we investigated a large number of various lipid metabolites in serum samples derived prior to conditioning therapy, and both the total levels of various lipid subclasses and the levels of individual biochemical/metabolites were included in our statistical analyses. Second, excessive postconditioning fluid overload and systemic pretransplant signs of ongoing inflammation have been identified as important pretransplant risk factors in allotransplant recipients, and acute GVHD is an important cause of nonrelapse mortality after allotransplantation. We therefore compared patients with and without fluid overload/inflammation/GVHD to identify lipid subclasses or individual metabolites with a potential impact on the posttransplant clinical course. Third, we used hierarchical clustering based on the identified lipid subclasses/individual biochemicals to identify subsets of recipients with similarities in their systemic pretransplant lipid profiles. Finally, we compared the identified patient subsets with regard to important clinical characteristics, including survival.

The lipid metabolites were classified into 16 main subclasses as shown in Table 6. However, it should be emphasized that lipid metabolites do not function as separate classes or subclasses; various lipid biochemicals show biological interactions across the subclasses [32,54,55,56,57,58,59,60,61] and they also show functional interactions with other metabolites, e.g., amino acid metabolism [47] and with various intracellular signaling proteins [62].

Several lipid metabolites are important regulators of both vascular, renal, endothelial and gastrointestinal functions as well as the functions of various immunocompetent cells (see Table S7). Pretransplant inflammation (immunocompetent cell activity) and early postconditioning excessive fluid overload (renal/vascular/endothelial function) predispose to acute GVHD which is an important cause of morbidity and mortality in allotransplant recipients [13]. Our present results show that both fluid overload, inflammation and acute GVHD are associated with altered systemic pretransplant levels of several lipid metabolites that are involved in the regulation of immunocompetent endothelial, and renal cells. Some of these metabolites probably reflect differences in systemic metabolic regulation, but a common characteristic of several of them is their important general functions in cell (surface) membranes both with regard to membrane structure, but also formation of lipid mediators and regulation of intracellular signaling (see Table S7).

The risk of severe morbidity and treatment-related mortality increases with age in allotransplant recipients [63], and aging is also associated with several metabolic modulations that can be reflected in the systemic metabolomic profile [64]. However, all our patients were relatively young, and we could not find any evidence for influence of aging on our identification of patient subsets based on the levels of individual lipid metabolites, and only a difference of borderline significance when comparing the total levels of biochemicals among lipid subsets (Figure 1, Table 4).

We compared the pretransplant lipidomic profiles for patients with and without pretransplant risk factors of decreased survival (i.e., signs of inflammation, fluid overload) and patients later developing acute GVHD. A large number of individual lipid metabolites differed significantly between patients with and without signs of inflammation, an increased number differed significantly also when comparing patients with and without later GVHD, whereas a relatively small number of metabolites differed for patients with and without fluid overload. However, almost all these individual biochemicals showed decreased levels for the affected patients, i.e., patients with inflammation/fluid overload/GVHD. Furthermore, the total levels of seven metabolite subclasses were also significantly decreased for patients with either inflammation, fluid overload or acute GVHD. We then performed a hierarchical cluster analysis based on the total concentrations, which furthermore identified a patient subset characterized by generally decreased lipid metabolite levels.

When analyzing the total serum concentrations of various lipid subclasses we detected a subset of patients with higher age, higher frequency of MDS or MDS-AML and more frequent signs of inflammation together with lower BMI and reduced levels of lipids belonging to several lipid subclasses. Signs of inflammation are associated with an adverse prognosis after allogeneic stem cell transplantation [13], and signs of inflammation are also common in MDS [65]. Differences in inflammatory regulation may also contribute to the advantage of using younger matched unrelated donors instead of matched sibling donors in elderly AML patients [66,67]. Furthermore, the effect of pretransplant ATG on non-relapse mortality seems to differ between patients with MDS and other hematological malignancies, an observation suggesting that the pretransplant immunobiology of MDS patients is different [68]. The overall survival of allotransplanted MDS patients has previously been relatively low compared with other patients [69]. The lipid metabolites associated with acute GVHD/inflammation/fluid overload function as regulators of inflammation/immunity as well as regulators of proliferation/survival of myeloid leukemic cells, and the MDS-associated lipidomic profiles may therefore be important for posttransplant complications/survival of allotransplanted MDS patients [42].

The BMI for the two contrasting patient subsets identified in Figure 1 differed significantly, and the patients in the upper MDS-associated cluster showing increased early death, also showed decreased pretransplant BMI. However, it should be emphasized that the median BMI for both groups was within the normal range according to the definitions suggested by World Health Organization [29]. The larger previous studies of the possible prognostic impact of BMI in allotransplant recipients have given conflicting results. First, a large registry Japanese study including 12,050 patients described decreased survival for underweight patients and this was due to an increased risk of relapse, whereas obesity was associated with increased non-relapse mortality [69,70]. A Swedish study also described increased transplant-related mortality and decreased overall- as well as relapse-free survival for patients with low BMI < 20 kg/m^2^ [71]. Furthermore, a Chinese study including 310 acute leukemia patients (177 with AML) also described decreased overall survival for patients with BMI below 23 kg/m^2^ [72]. However, two other studies including 384 [73] and 192 patients [74], respectively, could not detect any associations between survival and BMI. Possible explanations for these discrepancies could be differences with regard to ethnicity or patient inclusion/treatment [73]. Our present study suggests that BMI may be only another sign of a more complex metabolic high-risk phenotype that is associated with inflammation and has a prognostic impact that is mediated early after transplantation. An early prognostic impact is also suggested by a recent study describing no prognostic impact of the degree of BMI reduction during the posttransplant period [75]. A possible explanation for the general decrease in lipid biochemicals, low BMI and inflammation could also be nutritional problems due to the toxicity of pretransplant chemotherapy [76].

We did two different unsupervised hierarchical clustering analyses; the first was based on the total lipid concentrations that belong to seven lipid subclasses (Figure 1) and the second analysis on the levels of significantly altered individual lipid metabolites (Figure S2). These metabolites, used for hierarchical clustering, showed significant differences in three different patient comparisons, i.e., patients with and without pretransplant inflammation, early postconditional fluid overload or later development of steroid-requiring acute GVHD. Both cluster analyses showed that decreased systemic pretransplant levels of lipid metabolites were associated with pretransplant signs of inflammation and previous MDS. However, analysis of lipid metabolite subclass concentrations seems to reflect the recipient heterogeneity better than the analysis of individual lipid metabolite levels. Lipids belonging to the same biochemical subclasses usually clustered close to each other in the same main cluster or in a limited number of subclusters, showing that they showed similar variations between patients.

Taken together, our present results suggest that the pretransplant lipidomic profiles of allotransplant recipients should be further investigated as a possible prognostic target or as a tool to identify patient subsets that require different therapeutic interventions, e.g., nutritional support to normalize the profile. However, our study has several limitations. First, we investigated a relatively small group of patients. Second, we did not include a validation group, but we would then emphasize that our patients are unselected, i.e., it should be regarded as a population-based study and in our opinion, this strongly suggests that our results are representative. Third, future studies have to investigate whether the lipidomic profiles show similar alterations for patients allotransplanted for other malignant diseases or nonmalignant disorders. Additional studies are also needed to determine whether our present studies are representative for patients treated according to other regimens, e.g., transplanted with stem cell derived from matched unrelated donors, umbilical cord of haploidentical donors or pediatric recipients. We know that inflammation [13] and fluid overload [12] seem to have a prognostic impact for various allotransplanted patient subsets, and GVHD is also a general problem in allotransplant recipients [77]. Despite this, the immunobiology of various allotransplant recipients differ, e.g., GVHD is less important in umbilical cord transplantation [78] and the cytokine release syndrome is seen especially in haploidentical transplantation [79]. Finally, our analyses of posttransplant complications and survival should be interpreted with great care because differences between metabolic/lipidomic patient subsets can (at least partly) be caused by differences in conditioning therapy; this is true especially for the patient subsets identified in Figure 1.

MDS is associated with inflammatory complications [65], but despite this we did not find any significant association between pretransplant signs of inflammation and MDS/AML-MDS. This observation suggests that the risk of posttransplant acute GVHD/nonrelapse mortality associated with pretransplant inflammation [12,13] is similar for patients with and without MDS/MDS-AML. Furthermore, myeloablative busulfan-cyclophosphamide conditioning was used especially for patients regarded to have high-risk disease, including a majority of the patients with MDS-associated AML. MDS posttransplant relapse still remains the leading cause of failure after allotransplantation [80,81] and even posttransplant donor lymphocyte infusions are considered as a possible prophylactic strategy [81]. Busulfan-based conditioning is associated with a different risk of nonrelapse mortality in allotransplant recipients compared with other conditioning regimens [82,83,84,85,86]. For this reason, we did not include comparisons of posttransplant survival for the patient subsets identified in the cluster analysis based on the total levels of various lipid classes (Figure 1), because survival would then be reflected not only by differences in pretransplant lipid levels but possibly also by differences in conditioning therapy. In our opinion, the patient groups are too small to allow a reliable multivariate analysis of survival including both MDS, signs of inflammation and busulfan-based conditioning.

We identified two contrasting subsets of patients characterized by differences with regard to frequency of pretransplant MDS, signs of inflammation and early nonrelapse mortality (Table 4). Previous studies, as well as a large meta-analysis, have shown that pretransplant signs of inflammation are associated with decreased survival and increased acute GVHD [12,13]; an increased early mortality for this group is therefore not unexpected. Furthermore, even though inflammatory/immune complications are increased among MDS patients [65], we could not detect any difference in CRP levels between the patients with MDS/MDS-AML and the other patients. The frequency of GVHD (together with the risk of relapse) is regarded as a major challenge in allotransplanted MDS patients [81,87], and recent studies suggest that intensified GVHD prophylaxis should be considered for MDS patients [87,88]. However, our present observations suggest that this possible need for intensification is not due to an increased frequency of pretransplant inflammation among MDS patients.

Our present results suggest that the pretransplant lipidomic profiles differ between allotransplant recipients. Metabolic reprogramming is now suggested as a possible strategy for prophylaxis/treatment of GVHD [89,90,91,92,93], and our results suggest that the effect of such strategies will differ between patients and/or the optimal strategy for targeting metabolic pathways will differ between patients. Our study also suggests that pretransplant lipid levels may be used as biomarkers for the risk of GVHD and/or possibly also for subclassification of patients with regard to the possible and/or optimal use of metabolic targeting. The total levels of selected pretransplant lipid metabolite classes/subclasses seem to be useful for subclassification, and especially the levels of LPC, cholesteryl, LPE and monoacylglycerol esters (Table 5). However, future studies should also have a focus on the possible use of single metabolites, especially those metabolites showing strong associations with acute GVHD (Table S5).

The endothelial cell barrier is important both for regulation of chemotaxis/inflammation and for development of vascular leakage, and previous studies have demonstrated that adaptation of endothelial cell metabolism is important for modulation of endothelial function, e.g., the optimal barrier function and development of cancer-associated angiogenesis [94,95]. The pretransplant lipidomic status may therefore be important for or reflect abnormalities of endothelial functions; such abnormalities may also be important for fluid retention and/or inflammatory cell trafficking in allotransplant recipients. Furthermore, metabolic reprogramming of inflammatory/immunocompetent cells as GVHD prophylaxis/treatment may therefore have additional effects on inflammation/fluid balance through effects on endothelial cells even though relatively few lipid metabolites showed an association with fluid retention in our present study.

Metabolic alterations in intestinal epithelial cells and modulation of the gut microbiome can alter the risk of GVHD [96]; such mechanisms may also be important or be modulated by pretransplant anticancer chemotherapy and would thereby be reflected in the pretransplant lipidomic profiles. Previous murine studies have shown that metabolites (cholines, short-chain fatty acids, leukotrienes) derived from or modulated by the gut microbiota can aggravate GVHD through induction of proinflammatory macrophage polarization and/or modulation of T cell differentiation [97,98,99]. Immune homeostasis within the gut plays an important role in GVHD because gut microbiota and its metabolites impact gut integrity as well as inflammation and immune activation within the gut [98,100]. Such effects of the microbiota is further modulated by the nutritional intake [100]. Furthermore, the observation that donor T cells mediate GVHD through utilization of multiple metabolic pathways that can be distinct from the pathways used by regulatory T cells for their suppressive function, further support the hypothesis that metabolic targeting should be further explored as prophylaxis and/or treatment of GVHD [92,101]. Finally, several studies have demonstrated that ceramides function as intracellular mediators that are important in the regulation of inflammation and immunity [102,103,104,105]. However, previous studies have also shown that there are important functional/synthetic interactions between fatty acid synthesis, fatty acid uptake, as well as sphingolipid metabolism [54,56,59], and ceramides are involved in these interactions that are important for the regulation of inflammation [57,58,60,61]. Taken together, in our opinion these observations suggest that it is important to focus not only on single metabolites/metabolite subsets, but also on broader lipidomic profiles as well as the gut microbiota when investigating the role of lipids in GVHD.

## 5. Conclusions

As discussed above, pretransplant inflammation and early postconditional excessive fluid retention predispose to posttransplant non-relapse mortality in allotransplant recipients. Acute GVHD is an important (possibly the most important) cause of posttransplant non-relapse mortality. All of these three factors are associated with altered levels of various lipid metabolites that are important regulators of fundamental cellular functions, both in various normal cells and malignant cells. Metabolic targeting is now suggested as a possible therapeutic strategy in allotransplant recipients [89,90,91,92,93,106,107]. Our present study suggests that allotransplant recipients are heterogeneous with regard to their lipidomic profiles and their metabolic regulation, and the effect of metabolic targeting (e.g., nutritional support to increase/normalize the systemic lipid levels) and/or the optimal strategy for optimal targeting may therefore differ between patient subsets. We would also emphasize that several studies suggest that targeting the gut microbiome can also be a strategy for targeting metabolic pathways due to the absorption of metabolites derived from the gut microbiome.

## Figures and Tables

**Table 1:** Characteristics of the 92 patients (93 allotransplantations *) included in the study. Unless otherwise stated, the data are presented either as the number of patients or median (variation range) for the indicated parameter. One of the AML patients transplanted with detectable leukemia was treated according to the FLAMSA sequential treatment [24].

Characteristics of the 92 Patients (93 Allotransplantations *) Included in the Study			
**Demographic Data at Transplantation**		**Conditioning Therapy**	
Male/female patients	54/38	Busulfan + cyclophosphamide	52
Age (years, median and range)	54.5 (17–73)	Fludarabine + busulfan	21
Height (cm, median and range)	175 (149–197)	Fludarabine + treosulfan	16
Weight (kg, median and range)	71 (42–133)	Total body irradiation + cyclophosphamide	1
BMI (kg/m^2^, median and range)	23.3 (16.6–39.7)	Antithymocyte globulin + cyclophosphamide	1
		Fludarabine + busulfan + thiothepa	1
**Diagnosis**		FLAMSA	1
AML de novo	64		
AML with previous MDS	11	**Days until Three Consecutive Days above Indicated Level**	
MDS high-risk	18	Neutrophils > 0.2 × 10^9^/L	15 (10–29)
		Platelets > 20 × 10^9^/L	14 (9–35)
**Pretransplant Status for the AML Patients**			
CR1	58	Prognostic Parameters (Number of Patients)	
CR2	13	Early fluid overload (yes/no/not applicable)	37/52/4
CR3≥	2	Inflammation (yes/no)	36/57
No complete remission	2	Acute GVHD (yes/no/death before day +100 without acute GVHD)	32/51/10
**Pretransplant Hematological Status**			
White blood cell count (×10^9^/L)	3.6 (0.5–13.7)	**Death during Two Years of Follow-Up (Number of Patients)**	
Hb (g/dL)	10.4 (7.8–14.1)		
Platelets (×10^9^/L)	166 (6–779)	Overall death	54
CRP (mg/L)	6 (1–120)	Death within day +120 due to relapse	5
LDH (IU/dL)	181 (92–498)	Non-relapse death within day +120	12
**GVHD Prophylaxis**			
Methotrexate + cyclosporine	89		
Other cyclosporine-based regimens	4		

Abbreviations: AML, acute myeloid leukemia; BMI, body mass index; CR, complete hematological remission; CRP, C-reactive protein; GVHD, graft-versus-host disease; Hb, hemoglobin; LDH, lactate dehydrogenase; MDS, myelodysplastic syndrome; WBC, white blood cell count. * One patient diagnosed with AML was allotransplanted twice (93 allotransplants in total).

**Table 2:** The pretransplant systemic lipid profiles associated with posttransplant fluid overload, pretransplant systemic signs of inflammation (i.e., increased CRP levels) and posttransplant development of acute GVHD. For each of these three comparisons, the table presents (i) the number of patients with or without early fluid overload/pretransplant inflammation/acute GVHD (upper row) and (ii) the total number of lipid metabolites/biochemicals that differed significantly for each of the three comparisons (the middle row). The biochemicals that achieved statistical significance (i.e., *p* < 0.05) were identified by Welch’s two-sample *t*-test. We compared the total number of significantly altered metabolites for each of the three comparisons (fluid overload/inflammation/GVHD) with the corresponding expected number of differing metabolites that would be expected by coincidence, i.e., statistical significance defined by a *p* value of 0.05 and corresponding to 5% of the 960 detected/analyzed metabolites (corresponding to 48 metabolites). The Fisher’s exact test was used for these analyses, and the corresponding *p* values are indicated in parentheses in the second row). We also compared the number of significantly increased versus decreased metabolites with the distribution that would be expected by coincidence, and the corresponding *p* values after using the Fisher’s exact test for these comparisons are indicated in the bottom row).

Number of Patients in the Two Contrasting Groups for Each of the Three Comparisons	Fluid Overload (*n* = 37) No Fluid Overload (*n* = 52)	Systemic Inflammation (*n* = 36) No Inflammation (*n* = 57)	Acute GVHD (*n* = 32) No Acute GVHD (*n* = 51)
Total number of significantly altered metabolites (Fisher’s exact test, *p* values)	32 (not significant, *p* > 0.05)	117 (*p* < 0.00001)	69 (*p* = 0.0382)
The number of significantly increased (↑) versus decreased (↓) metabolites (Fisher’s exact test, *p* values)	↑0 ↓32 (*p* < 0.00001)	↑1 ↓116 (*p* < 0.00001)	↑1 ↓69 (*p* < 0.00001)

**Table 3:** The differences in serum lipid profiles between allotransplant recipients with and without preconditioning signs of inflammation or later development of acute GVHD; a summary of the overall results. The table presents the *p* values for those seven out of 14 investigated lipid classes/subclasses that showed statistically significant differences when comparing their total levels for patients with and without inflammation and acute GVHD. A more detailed presentation showing the number of significantly altered metabolites for each of the two comparisons (i.e., with and without inflammation/GVHD, respectively), together with the corresponding *r* values is given in Table S1.

Main Group of Lipids	Lipid Subset	Inflammation	Acute GVHD
		*p* Value	*p* Value
Lysophosphatidylcholine	LPC Ester	0.0001	
Lysophosphatidylethanolamine	LPE Ester	0.0491	
Phosphatidylinositol	PI Ester	0.0315	
Cholesteryl Ester	CE Ester	0.0197	
Sphingolipids	Lactosylceramide		0.0227
Diacylglycerols	DAG ester		0.0335
Monoacylglycerols	MAG Ester		0.0158

Total serum levels were determined for the 14 lipid classes phosphatidylcholines, lysophosphatidylcholines (LPC), phosphatidylethanolamines, lysophosphatidylethanolamines (LPE), phosphatidylinositols (PI), ceramides, dihydroceramides, hexosylceramides, lactosylceramides, sphingomyelins, cholesteryl esters (CE), diacyglycerols (DAG), triacylglycerols and monoacylglycerols.

**Table 4:** A comparison of the two contrasting patient subsets identified in the hierarchical cluster analysis of total lipid levels (from Figure 1), and a comparison of the upper main subcluster (the 24 patients indicated by purple color in the right part of Figure 1, with generally low levels of lipids) and the lower subcluster (the 25 patients indicated by brown color to the right in the lowest part of Figure 1 and with generally high levels of lipids). For categorized data, we used the Fisher’s test, and for continuous data we used the Mann–Whitney U test for comparison of these two patient subsets; the *p* values are indicated in the right column of the table.

Patient Parameter	Upper Patient Subset (Generally Low Lipid Levels); The Upper Subcluster Including 24 Patients	Lower Patient Subset (Generally High Lipid Levels); The Lower Subcluster Including 25 Patients	*p* Value
Median age (range)	56 years (34–66 years)	48 years (21–71 years)	0.031
Previous MDS	12 patients	3 patients	0.0054
Inflammation (increased pretransplant CRP)	16 patients	4 patients	0.0004
Excessive early fluid overload	11 patients	8 patients	0.37
Busulfan-containing conditioning therapy	23 patients	12 patients	0.0003
Steroid-requiring acute GVHD	11 patients	6 patients	0.068
Non-relapse mortality before day +120 *	6 patients	1 patient	0.042

* Four patients in the upper main cluster and two patients in the lower subcluster died from early relapse before day +120 and were therefore excluded from the statistical analysis.

**Table 5:** A comparison of the two contrasting patient subsets identified in the hierarchical cluster analysis of total lipid levels (from Figure 1), and a comparison of the upper main subcluster (the 24 patients with generally low levels of lipids indicated by purple color at the top right part of Figure 1) and the lower subcluster (the 25 patients with generally high levels of lipids indicated by brown color at the right, lower part of Figure 1). We compared the total levels of the seven lipid classes/subclasses included in the clustering analysis. All concentrations are given in μM, and the Mann–Whitney U test was used for the statistical comparisons of these two contrasting patient subsets; the corresponding *p* values are shown in the far right column.

Main Lipid Class	Lipid Subclass	Upper Patient Subset; The Upper Main Cluster Including 24 Patients	Lower Patient Subset; The Lower Main Cluster Including 25 Patients	*p* Value
Lysophosphatidylcholine	LPC Ester	141 (70–215)	306 (194–306)	<0.00001
Lysophosphatidylethanolamine	LPE Ester	3.5 (1.5–4.6)	6.7 (4.0–16.2)	0.0032
Phosphatidylinositol	PI Ester	6.8 (3.6–12.7)	11.6 (5.6–18.5)	0.0088
Cholesteryl Ester	CE Ester	1866 (1042–3025)	2640 (1745–3870)	<0.00001
Sphingolipids	Lactosylceramide	1.4 (0.6–2.3)	1.8 (1.1–4.0)	0.43
Diacylglycerol	DAG Ester	29.8 (8.6–29.8)	53.3 (14.3–118)	0.48
Monoacylglycerol	MAG Ester	2.3 (0.9–75.7)	11.7 (1.3–143)	0.0028

**Table 6:** The number and classification of lipid species showing significantly different levels when comparing patients with and without pretransplant inflammation, early postconditioning fluid overload and posttransplant acute GVHD. The table presents the total number of analyzed metabolites for each lipid subclass and the number of differentially expressed metabolites that were altered within the different comparing groups (with and without inflammation/fluid overload/GVHD). A simplified visualization of these results is presented in Figure S1.

Main Lipid Class	Lipid Subclass	Number of Analyzed Metabolites	Pretransplant Inflammation	Early Fluid Overload	Acute GVHD
Phosphatidylcholine	PC Ester	114	25	7	5
Lysophosphatidylcholine	LPC Ester	18	18	0	0
Phosphatidylethanolamine	PE Ester	63	3	2	0
	PE Ether	28	9	0	0
	PE Plasmalogen	40	20	1	1
Lysophosphatidylethanolamine	LPE Ester	15	2	2	1
Phosphatidylinositol	PI Ester	23	5	6	1
Cholesteryl Ester	CE Ester	26	13	4	0
Sphingolipids	Ceramide	12	3	0	0
	Dihydroceramide	14	2	1	1
	Hexosylceramide	12	0	0	1
	Lactosylceramide	12	5	2	2
	Sphingomyelin	12	3	0	0
Monoacylglycerol	MAG Ester	26	1	0	16
Diacylglycerol	DAG Ester	58	0	1	28
Triacylglycerol	TAG Ester	518	8	6	13

**Table 7:** The classification of lipid metabolites showing significantly different levels when comparing patients with and without pretransplant inflammation, early postconditioning fluid overload and posttransplant acute GVHD. The table shows the number of different lipid metabolites (and their percentages) in the two main clusters identified in the unsupervised hierarchical cluster analysis, based on the lipid metabolites that differed significantly between patients with and without inflammation/fluid overload/acute GVHD (see Figure S2). A more detailed presentation showing the number of metabolites found in each of the six subclusters is presented in Table S6.

Lipid Main Class	Lipid Subclass	Cluster 1 (103 Lipid Metabolites)	Cluster 2 (66 Lipid Metabolites)
Phosphatidylcholine	PC Ester	12 (12%)	14 (21%)
Lysophosphatidylcholine	LPC Ester		17 (25%)
Phosphatidylethanolamine	PE Ester/Ether/ Plasmalogen	11 (11%)	16 (24%)
Lysophosphatidylethanolamine	LPE Ester	1 (1%)	2 (3%)
Phosphatidylinositol	PI Ester	2 (2%)	3 (5%)
Cholesteryl Ester	CE Ester	13 (13%)	3 (5%)
Sphingolipids	Ceramide	3 (3%)	
	Dihydroceramide	2 (2%)	
	Hexosylceramide	1 (1%)	
	Lactosylceramide		6 (9%)
	Sphingomyelin	3 (3%)	
Monoacylglycerol	MAG Ester	17 (16%)	
Diacylglycerol	DAG Ester	28 (27%)	1 (2%)
Triacylglycerol	TAG Ester	14 (13%)	4 (6%)

## Data Availability

The data presented in this study are available on request from the corresponding author.

## Supplementary Materials

The following are available online at https://www.mdpi.com/article/10.3390/cancers14122910/s1. Expanded methods: The Metabolon platform. Figure S1: The lipid subclasses with significantly altered levels when comparing patients with and without pretransplant inflammation, early postconditioning fluid overload and posttransplant acute GVHD. Figure S2: Hierarchical clustering of lipid biochemicals. Table S1: Differences in serum lipid profiles between allotransplant recipients with and without preconditioning signs of inflammation or later development of acute GVHD. Table S2: A summary of metabolites showing significantly different levels when comparing patients with and without pretransplant signs of inflammation (i.e., increased serum CRP levels). Table S3: A summary of metabolites showing significantly different levels when comparing patients with and without early postconditional fluid overload. Table S4. The metabolites showing statistically significant differences (Welch’s two sample *t*-test, *p* < 0.05) when comparing patients with versus without inflammation/fluid retention/acute GVHD. Table S5. A summary of metabolites showing significantly different levels when comparing patients with and without steroid-requiring posttransplant acute GVHD. Table S6. The classification of lipid metabolites showing significantly different levels when comparing patients with and without pretransplant inflammation, early postconditioning fluid overload and posttransplant acute GVHD. Table S7. The differences in serum lipid profiles between allotransplant recipients with and without preconditioning signs of inflammation; a summary of important biological characteristics for lipid biochemical subclasses investigated in the present study. References [30,32,33,34,35,36,37,38,39,40,41,42,43,44,47,54,55,56,57,58,59,60,61,62,108,109,110,111,112,113,114,115,116,117,118,119,120,121,122,123,124,125,126] are cited in the supplementary materials.
