# Supplementary material for: Pretransplant Systemic Lipidomic Profiles in Allogeneic Stem Cell Transplant Recipients
Source: Cancers (Basel). 2022 Jun 13;14(12):2910. doi: 10.3390/cancers14122910 (PMC9220974; doi:10.3390/cancers14122910)
Supplement: Supplementary file 1 [file cancers-14-02910-s001.zip › cancers-1705328-supplementary.pdf]

cancers

Supplementary Material

Pretransplant systemic lipidomic profiles in allogeneic stem cell transplant recipients

Kimberley Joanne Hatfield [1], Øystein Bruserud [2,3]* and Håkon Reikvam [2,3]
* Correspondence: oystein.bruserud@helse-bergen.no

Expanded Methods:

i. **The Metabolon Platform**

Supplementary Figures

ii. **Figure S1. The l**ipid subclasses with significantly altered levels when comparing patients with and without pretransplant inflammation, early postconditioning fluid overload and posttransplant acute GVHD.
iii. **Figure S2.** Hierarchical clustering of lipid biochemicals.

Supplementary Tables

iv. **Table S1.** Differences in serum lipid profiles between allotransplant recipients with and without preconditioning signs of inflammation or later development of acute GVHD.
v. **Table S2.** A summary of metabolites showing significantly different levels when comparing patients with and without pretransplant signs of inflammation (i.e., increased serum CRP levels).
vi. **Table S3.** A summary of metabolites showing significantly different levels when comparing patients with and without early post-conditional fluid overload.
vii. **Table S4. The m**etabolites showing statistically significant differences (Welch's two sample t-test, $p<0.05$) when comparing patients with versus without inflammation/fluid retention/acute GVHD.
viii. **Table S5.** A summary of metabolites showing significantly different levels when comparing patients with and without steroid-requiring posttransplant acute GVHD.
ix. **Table S6. The c**lassification of lipid metabolites showing significantly different levels when comparing patients with and without pretransplant inflammation, early postconditioning fluid overload and posttransplant acute GVHD.
x. **Table S7. The d**ifferences in serum lipid profiles between allotransplant recipients with and without preconditioning signs of inflammation; a summary of important biological characteristics for lipid biochemical subclasses investigated in the present study.

References

1. Expanded Methods: The Metabolon Platform

Sample Accessioning. Following receipt, samples were inventoried and immediately stored at -80°C until processed.

Sample Preparation. Samples were prepared using the automated MicroLab STAR® system from Hamilton Company. Several recovery standards were added prior to the first step in the extraction process for QC purposes. To remove protein, dissociate small molecules bound to protein or trapped in the precipitated protein matrix, and to recover chemically diverse metabolites, proteins were precipitated with methanol under vigorous shaking for 2 minutes followed by centrifugation. The resulting extract was divided into five fractions: two for analysis by two separate reverse phase (RP)/UPLC-MS/MS methods with positive ion mode electrospray ionization (ESI), one for analysis by RP/UPLC-MS/MS with negative ion mode ESI, one for analysis by HILIC/UPLC-MS/MS with negative ion mode ESI, and one sample was reserved for backup. Samples were placed briefly on a TurboVap® (Zymark) to remove the organic solvent. The sample extracts were stored overnight under nitrogen before preparation for analysis.

Several types of controls were analyzed in concert with the experimental samples: a pooled matrix sample generated by using of a pool of well-characterized human plasma served as a technical replicate throughout the data set; extracted water samples served as process blanks; and a cocktail of quality control standards that were carefully chosen not to interfere with the measurement of endogenous compounds were spiked into every analyzed sample, allowed instrument performance monitoring and aided chromatographic alignment. Instrument variability was determined by calculating the median relative standard deviation (RSD) for the standards that were added to each sample prior to injection into the mass spectrometers. Overall process variability was determined by calculating the median RSD for all endogenous metabolites (i.e., non-instrument standards) present in 100% of the pooled matrix samples. Experimental samples were randomized across the platform run with quality control samples spaced evenly among the injections.

Ultrahigh Performance Liquid Chromatography-Tandem Mass Spectroscopy (UPLC-MS/MS). All methods utilized a Waters ACQUITY ultra-performance liquid chromatography (UPLC) and a Thermo Scientific Q-Exactive high resolution/accurate mass spectrometer interfaced with a heated electrospray ionization (HESI-II) source and Orbitrap mass analyzer operated at 35,000 mass resolution. The sample extract was dried then reconstituted in solvents compatible to each of the four methods. Each reconstitution solvent contained a series of standards at fixed concentrations to ensure injection and chromatographic consistency. One aliquot was analyzed using acidic positive ion conditions, chromatographically optimized for more hydrophilic compounds. In this method, the extract was gradient eluted from a C18 column (Waters UPLC BEH C18-2.1x100 mm, 1.7 µm) using water and methanol, containing 0.05% perfluoropentanoic acid (PFPA) and 0.1% formic acid (FA). Another aliquot was also analyzed using acidic positive ion conditions; however, it was chromatographically optimized for more hydrophobic compounds. In this method, the extract was gradient eluted from the same afore mentioned C18 column using methanol, acetonitrile, water, 0.05% PFPA and 0.01% FA and was operated at an overall higher organic content. Another aliquot was analyzed using basic negative ion optimized conditions using a separate dedicated C18 column. The basic extracts were gradient eluted from the column using methanol and water, however with 6.5mM Ammonium Bicarbonate at pH 8. The fourth aliquot was analyzed via negative ionization following elution from a HILIC column (Waters

UPLC BEH Amide 2.1x150 mm, 1.7 µm) using a gradient consisting of water and acetonitrile with 10mM Ammonium Formate, pH 10.8. The MS analysis alternated between MS and data-dependent MS^n scans using dynamic exclusion. The scan range varied slightly between methods but covered 70-1000 m/z. Raw data files are archived and extracted as described below.

Bioinformatics. The informatics system consisted of four major components, the Laboratory Information Management System (LIMS), the data extraction and peak-identification software, data processing tools for QC and compound identification, and a collection of information interpretation and visualization tools for use by data analysts. The hardware and software foundations for these informatics components were the LAN backbone and a database server running Oracle 10.2.0.1 Enterprise Edition.

LIMS. The purpose of the Metabolon LIMS system was to enable fully auditable laboratory automation through a secure, easy to use, and highly specialized system. The scope of the Metabolon LIMS system encompasses sample accessioning, sample preparation and instrumental analysis and reporting and advanced data analysis. All of the subsequent software systems are grounded in the LIMS data structures. It has been modified to leverage and interface with the in-house information extraction and data visualization systems, as well as third party instrumentation and data analysis software.

Data Extraction and Compound Identification. Raw data was extracted, peak-identified and QC processed using Metabolon's hardware and software. Compounds were identified by comparison to library entries of purified standards or recurrent unknown entities. Metabolon maintains a library based on authenticated standards that contains the retention time/index (RI), mass to charge ratio (*m/z)*, and chromatographic data (including MS/MS spectral data) on all molecules present in the library. Furthermore, biochemical identifications are based on three criteria: retention index within a narrow RI window of the proposed identification, accurate mass match to the library +/- 10 ppm, and the MS/MS forward and reverse scores between the experimental data and authentic standards. The MS/MS scores are based on a comparison of the ions present in the experimental spectrum to the ions present in the library spectrum. While there may be similarities between these molecules based on one of these factors, the use of all three data points can be utilized to distinguish and differentiate biochemicals. More than 3300 commercially available purified standard compounds have been acquired and registered into LIMS for analysis on all platforms for determination of their analytical characteristics. Additional mass spectral entries have been created for structurally unnamed biochemicals, which have been identified by virtue of their recurrent nature (both chromatographic and mass spectral).

Curation. A variety of curation procedures were carried out to ensure that a high-quality data set was made available for statistical analysis and data interpretation. The QC and curation processes were designed to ensure accurate and consistent identification of true chemical entities, and to remove those representing system artifacts, misassignments, and background noise. Metabolon data analysts use proprietary visualization and interpretation software to confirm the consistency of peak identification among the various samples. Library matches for each compound were checked for each sample and corrected if necessary.

Metabolite Quantification and Data Normalization. Peaks were quantified using area-under-the-curve. For studies spanning multiple days, a data normalization step was performed to correct variation resulting from instrument inter-day tuning differences. Essentially, each compound was corrected in

run-day blocks by registering the medians to equal one (1.00) and normalizing each data point proportionately (termed the "block correction"). For studies that did not require more than one day of analysis, no normalization is necessary, other than for purposes of data visualization. In certain instances, biochemical data may have been normalized to an additional factor (e.g., cell counts, total protein as determined by Bradford assay, osmolality, etc.) to account for differences in metabolite levels due to differences in the amount of material present in each sample.

Complex Lipid Platform. Lipids were extracted from the serum samples in the presence of deuterated internal standards using an automated BUME extraction according to the method of Lofgren et al [1]. The extracts were concentrated under nitrogen and reconstituted in 0.25mL of 10mM ammonium acetate dichloromethane:methanol (50:50). The extracts were transferred to inserts and placed in vials for infusion-MS analysis, performed on a Shimazdu LC with nano PEEK tubing and the Sciex SelexIon-5500 QTRAP. The samples were analyzed via both positive and negative mode electrospray. The 5500 QTRAP scan was performed in MRM mode with a total of more than 1100 MRMs. Individual lipid species were quantified by taking the peak area ratios of target compounds and their assigned internal standards, then multiplying by the concentration of internal standard added to the sample. Lipid species concentrations were background-subtracted using the concentrations detected in process blanks (water extracts) and run day normalized (when applicable). The resulting background-subtracted, run-day normalized lipid species concentrations were then used to calculate the lipid class and fatty acid total concentrations, as well as the mol% composition values for lipid species, lipid classes, and fatty acids.

Metabolon's Complex Lipid Panel (CLP)

Metabolon's Complex Lipid Panel utilizes three key technologies to achieve broad coverage, quantification, and specificity in lipidomic analysis. These are flow injection analysis (FIA), differential mobility spectrometry (DMS), and multiple reaction monitoring (MRM). Flow injection analysis refers to the fact that the lipid sample solution is continuously infused into the mass spectrometer without the use of any chromatographic column. FIA is used because chromatographic separation of lipids is extremely challenging and can actually hinder quantification if a lipid elutes from the column at a different time, and in the presence of different interferences, than its internal standard. With FIA, a uniform sample is analyzed throughout the infusion (typically ~6 minutes in the case of the CLP), allowing multiple replicate measurements for more robust and reproducible results. The DMS technology takes advantage of the fact that a molecule's trajectory through an electric field is affected by its size, shape and dipole moment. After ionization in the source of the mass spectrometer, lipids are introduced into the SelexIon DMS cell. The DMS cell acts as a lipid filter or gate that permits a specific lipid class to pass into the mass spectrometer, while the other lipid classes are filtered out. This technology removes the isomeric lipid interferences between lipid classes, permitting more specific identification of all detected lipid species. The DMS cell cycles through the different lipid classes over the course of a single sample infusion, sequentially passing each lipid class into the mass spectrometer for analysis. After exiting the DMS cell, the lipids from the selected lipid class enter the mass spectrometer for MRM analysis. MRM analysis requires a triple quadrupole mass spectrometer, in which the first and third quadrupole can filter the individual lipid species on the basis of mass-to-charge ratio (*m*/*z*). In an MRM analysis, the first quadrupole (Q1) filters on the basis of the *m*/*z* of the intact lipid species, while the third quadrupole (Q3) filters on the basis of the *m*/*z* of a characteristic fragment of that same lipid species, such as one of the fatty acid side chains. MRM analysis is the standard methodology

used for mass spectrometry-based clinical assays, as it is the most quantitatively accurate and provides a high degree of analyte specificity. Together, the DMS cell and the MRM analysis ensure that the signal that is finally recorded by the detector specifically and accurately measures an individual lipid species.

cancers

Figure S1. The lipid subclasses with significantly altered levels when comparing patients with and without pretransplant inflammation, early postconditioning fluid overload and posttransplant acute GVHD. The table indicates the levels of differentially expressed lipids/biochemicals (i.e. grey color indicates classes with significant changes for at least 25% of the analyzed metabolites; white boxes/color indicate significant changes for less than 25% of the analyzed metabolites.) for each lipid subclass among the total number of biochemicals that were analysed for each lipid subclass; a comparison of the different groups (with and without inflammation/fluid overload/GVHD).

**Main lipid class**	**Lipid subclass**	**Pretransplant inflammation**	**Early fluid overload**	**Acute GVHD**
Phosphatidylcholine	PC Ester			
Lysophosphatidylcholine	LPC Ester			
Phosphatidylethanolamine	PE Ester			
	PE Ether			
	PE Plasmalogen			
Lysophosphatidylethanolamine	LPE Ester			
Phosphatidylinositol	PI Ester			
Cholesteryl Ester	CE Ester			
Sphingolipids	Ceramide			
	Dihydroceramide			
	Hexosylceramide			
	Lactosylceramide			
	Sphingomyelin			
Monoacylglycerol	MAG Ester			
Diacylglycerol	DAG Ester			
Triacylglycerol	TAG Ester			

At least 75 % of the analyzed biochemicals were significantly altered and/or at least 25 of the analyzed biochemicals.

At least 50 % of the analyzed biochemicals were significantly altered.

At least 25 % of the analyzed biochemicals were significantly altered.

cancers

Figure S2: Hierarchical clustering of lipid biochemicals. The expression levels of significantly altered lipid biochemicals were used for hierarchical cluster analysis. We identified two main clusters based upon analysis of individual lipid metabolites, referred to as clusters 1 and 2, and each of these two main clusters could be further divided into three subclusters 1.1/1.2/1.3 and 2.1/2.2/2.3, respectively (lower part). The distribution of various lipid metabolites into these six subclusters is demonstrated in Table 7 (green color indicates low levels, purple indicates high levels). Patient subsets are shown at the far right and patients with or without fluid overload (capillary leak), signs of inflammation, and/or aGVHD are indicated in the columns to the far right.

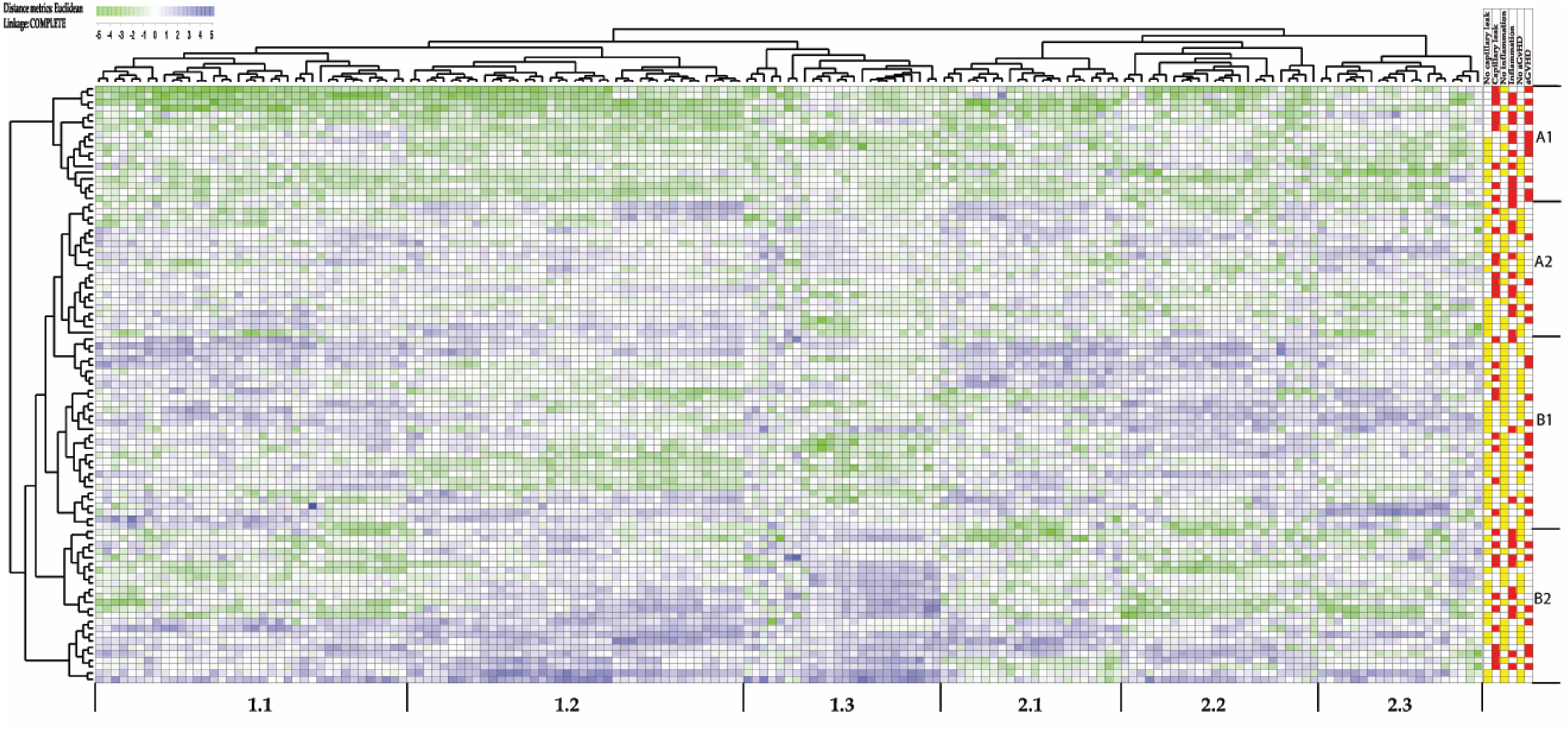

cancers

Table S1. Differences in serum lipid profiles between allotransplant recipients with and without preconditioning signs of inflammation or later development of acute GVHD. The table presents the significant differences and correlation coefficients (*p*/*r* values) for those seven out of 14 investigated lipid classes/subclasses that showed statistically significant differences when comparing their total levels for patients with and without inflammation and acute GVHD. The table shows the number of individual biochemicals/metabolites that showed statistically significant differences when comparing patients with and without inflammation/acute GVHD (i.e. significant metabolites/total number of metabolites in the subclass).

Main group	Biochemical subset	Inflammation		Acute GVHD	
		Number	*p*/*r* value	Number	*p*/*r* value
Lysophosphatidylcholine	LPC Ester	18/18	0.0001/0.0198		
Lysophosphatidylethanolamine	LPE Ester	2/15	0.0491/0.2692	1/15	
Phosphatidylinositol	PI Ester	5/23	0.0315/0.2140	1/23	
Cholesteryl Ester	CE Ester	13/25	0.0197/0.1658		
Sphingolipids	Lactosylceramide	5/12		2/12	0.0227/0.1861
Diacylglycerols	DAG ester			28/58	0.0335/0.1861
Monoacylglycerols	MAG Ester	1/26		16/26	0.0158/0.1861

Total serum levels were determined for the 14 lipid classes: phosphatidylcholines, lysophosphatidylcholines (LPC), phosphatidylethanolamines, lysophosphatidylethanolamines (LPE), phosphatidylinositols (PI), ceramides, dihydroceramides, hexosylceramides, lactosylceramides, sphingomyelins, cholesteryl esters (CE), diacyglycerols (DAG), triacylglycerols and monoacylglycerols (MAG).

cancers

Table S2. A summary of metabolites showing significantly different levels when comparing patients with and without pretransplant signs of inflammation (i.e., increased serum CRP levels). The name/identity of metabolites along with the classification is shown, the Human Metabolome Database (HMDB link), ratio, *p* value and *q* value for each individual metabolite. The ratio represents the median level for patients with versus patients without inflammation (green color indicates ratio <1.00, red color indicates ratio >1.00).

Biochemical name	Super pathway	Subpathway	HMDB	Ratio	*p* value	*q* value
PE(O-16:0/20:3)	Phosphatidylethanolamine	PE Ether		0.65	0.0000	0.0003
LPC(14:0)	Lysophosphatidylcholine	LPC Ester	HMDB10379	0.73	0.0001	0.0156
LPC(17:0)	Lysophosphatidylcholine	LPC Ester		0.73	0.0001	0.0156
TAG55:8-FA20:4	Triacylglycerol	TAG Ester		0.10	0.0001	0.0198
LPC(18:0)	Lysophosphatidylcholine	LPC Ester	HMDB10384	0.71	0.0002	0.0217
LPC(16:0)	Lysophosphatidylcholine	LPC Ester	HMDB10382	0.74	0.0004	0.0344
LPC(15:0)	Lysophosphatidylcholine	LPC Ester	HMDB10381	0.77	0.0005	0.0344
TAG53:6-FA18:1	Triacylglycerol	TAG Ester		0.40	0.0005	0.0344
LPC(16:1)	Lysophosphatidylcholine	LPC Ester	HMDB10383	0.76	0.0006	0.0381
LPC(20:1)	Lysophosphatidylcholine	LPC Ester	HMDB10391	0.74	0.0006	0.0381
LPC(18:2)	Lysophosphatidylcholine	LPC Ester		0.74	0.0008	0.0423
LPC(18:1)	Lysophosphatidylcholine	LPC Ester		0.76	0.0009	0.0423
LPC(20:0)	Lysophosphatidylcholine	LPC Ester	HMDB10390	0.72	0.0009	0.0423
PC(20:0/20:4)	Phosphatidylcholine	PC Ester	HMDB08279	0.75	0.0011	0.0496
LPC(20:2)	Lysophosphatidylcholine	LPC Ester	HMDB10392	0.73	0.0012	0.0513
PE(18:2/20:4)	Phosphatidylethanolamine	PE Ester	HMDB09102	0.73	0.0014	0.0565
PC(18:2/20:5)	Phosphatidylcholine	PC Ester	HMDB08149	0.71	0.0017	0.0565
PE(P-18:0/18:1)	Phosphatidylethanolamine	PE Plasmalogen	HMDB11375	0.74	0.0017	0.0565
PE(P-18:0/20:5)	Phosphatidylethanolamine	PE Plasmalogen	HMDB11387	0.65	0.0017	0.0565
PE(O-16:0/18:2)	Phosphatidylethanolamine	PE Ether		0.75	0.0022	0.0650
PE(P-18:0/16:0)	Phosphatidylethanolamine	PE Plasmalogen	HMDB11371	0.77	0.0022	0.0650
PE(P-18:0/18:3)	Phosphatidylethanolamine	PE Plasmalogen	HMDB11378	0.77	0.0023	0.0650
PE(P-18:1/20:5)	Phosphatidylethanolamine	PE Plasmalogen	HMDB11420	0.68	0.0028	0.0768
PC(16:0/20:1)	Phosphatidylcholine	PC Ester	HMDB07978	0.83	0.0030	0.0774
PE(P-16:0/18:2)	Phosphatidylethanolamine	PE Plasmalogen	HMDB11343	0.73	0.0030	0.0774
PE(P-18:0/18:2)	Phosphatidylethanolamine	PE Plasmalogen	HMDB11376	0.75	0.0038	0.0946
MAG(22:1)	MAG	Ester		0.81	0.0040	0.0956
CE(12:0)	Cholesterol Ester	CE Ester	HMDB02262	0.61	0.0043	0.0988
PE(P-18:0/20:3)	Phosphatidylethanolamine	PE Plasmalogen	HMDB11384	0.74	0.0050	0.1046
PE(P-18:1/18:1)	Phosphatidylethanolamine	PE Plasmalogen	HMDB11441	0.77	0.0050	0.1046
PI(18:1/18:1)	Phosphatidylinositol	PI Ester	HMDB09837	0.74	0.0050	0.1046
PE(P-18:0/20:4)	Phosphatidylethanolamine	PE Plasmalogen	HMDB11386	0.75	0.0055	0.1048
SM(22:1)	Sphingolipids	Sphingomyelin	HMDB12104	0.85	0.0055	0.1048
PE(18:2/16:1)	Phosphatidylethanolamine	PE Ester	HMDB09089	0.75	0.0056	0.1048
CE(14:0)	Cholesterol Ester	CE Ester	HMDB06725	0.77	0.0057	0.1048
PE(O-16:0/20:5)	Phosphatidylethanolamine	PE Ether		0.79	0.0061	0.1063
PI(18:0/20:3)	Phosphatidylinositol	PI Ester	HMDB09814	0.77	0.0061	0.1063
PE(P-16:0/20:3)	Phosphatidylethanolamine	PE Plasmalogen	HMDB11351	0.72	0.0063	0.1063
CE(18:0)	Cholesterol Ester	CE Ester	HMDB10368	0.85	0.0064	0.1063

LPC(22:4)	Lysophosphatidylcholine	LPC Ester	HMDB10401	0.87	0.0068	0.1075
DCER(24:0)	Sphingolipids	Dihydroceramide	HMDB11768	0.83	0.0068	0.1075
CER(14:0)	Sphingolipids	Ceramide	HMDB11773	0.84	0.0070	0.1075
PE(O-18:0/16:0)	Phosphatidylethanolamine	PE Ether		0.93	0.0072	0.1090
LPC(18:3)	Lysophosphatidylcholine	LPC Ester	HMDB10388	0.79	0.0075	0.1113
PE(O-16:0/20:4)	Phosphatidylethanolamine	PE Ether		0.75	0.0079	0.1142
PC(12:0/18:1)	Phosphatidylcholine	PC Ester		0.77	0.0082	0.1148
PC(18:2/20:4)	Phosphatidylcholine	PC Ester	HMDB08147	0.81	0.0083	0.1148
LCER(22:0)	Sphingolipids	Lactosylceramide	HMDB11594	0.86	0.0086	0.1164
PC(18:2/22:6)	Phosphatidylcholine	PC Ester	HMDB08156	0.83	0.0088	0.1164
PE(P-18:0/22:6)	Phosphatidylethanolamine	PE Plasmalogen	HMDB11394	0.77	0.0094	0.1226
PC(17:0/22:5)	Phosphatidylcholine	PC Ester		0.80	0.0099	0.1242
LPC(22:5)	Lysophosphatidylcholine	LPC Ester	HMDB10403	0.81	0.0100	0.1242
LCER(24:0)	Sphingolipids	Lactosylceramide	HMDB11595	0.82	0.0102	0.1242
PC(14:0/20:3)	Phosphatidylcholine	PC Ester	HMDB07882	0.76	0.0103	0.1242
LPC(20:4)	Lysophosphatidylcholine	LPC Ester	HMDB10395	0.78	0.0107	0.1264
LPC(20:3)	Lysophosphatidylcholine	LPC Ester	HMDB10394	0.79	0.0113	0.1312
PC(18:2/18:2)	Phosphatidylcholine	PC Ester	HMDB08138	0.76	0.0115	0.1312
LPE(16:0)	Lysophosphatidylethanolamine	LPE Ester	HMDB11503	0.82	0.0120	0.1351
LPE(18:0)	Lysophosphatidylethanolamine	LPE Ester	HMDB11130	0.80	0.0122	0.1351
PE(P-16:0/18:1)	Phosphatidylethanolamine	PE Plasmalogen	HMDB11342	0.80	0.0124	0.1352
CE(17:0)	Cholesterol Ester	CE Ester	HMDB60059	0.84	0.0129	0.1358
LCER(22:1)	Sphingolipids	Lactosylceramide	HMDB11594	0.85	0.0129	0.1358
CE(18:1)	Cholesterol Ester	CE Ester	HMDB00918	0.87	0.0136	0.1413
PE(P-18:1/20:3)	Phosphatidylethanolamine	PE Plasmalogen	HMDB11450	0.78	0.0138	0.1413
PC(14:0/20:4)	Phosphatidylcholine	PC Ester	HMDB07883	0.82	0.0144	0.1453
PC(18:0/14:0)	Phosphatidylcholine	PC Ester	HMDB08031	0.83	0.0153	0.1511
PE(P-16:0/20:5)	Phosphatidylethanolamine	PE Plasmalogen	HMDB11354	0.72	0.0155	0.1513
PE(P-18:1/18:2)	Phosphatidylethanolamine	PE Plasmalogen	HMDB11442	0.78	0.0159	0.1513
PE(P-16:0/20:4)	Phosphatidylethanolamine	PE Plasmalogen	HMDB11353	0.78	0.0160	0.1513
LPC(20:5)	Lysophosphatidylcholine	LPC Ester	HMDB10397	0.81	0.0171	0.1590
TAG53:7-FA18:3	Triacylglycerol	TAG Ester		0.66	0.0172	0.1590
CE(22:0)	Cholesterol Ester	CE Ester	HMDB06727	0.79	0.0176	0.1594
PC(20:0/20:5)	Phosphatidylcholine	PC Ester	HMDB08281	0.77	0.0178	0.1594
TAG55:7-FA20:3	Triacylglycerol	TAG Ester		0.43	0.0180	0.1594
PE(P-18:0/22:5)	Phosphatidylethanolamine	PE Plasmalogen	HMDB11393	0.81	0.0185	0.1615
LCER(26:0)	Sphingolipids	Lactosylceramide	HMDB04874	0.85	0.0196	0.1658
PE(P-18:1/16:0)	Phosphatidylethanolamine	PE Plasmalogen	HMDB11437	0.80	0.0197	0.1658
CE(18:2)	Cholesterol Ester	CE Ester	HMDB00610	0.88	0.0221	0.1787
PC(15:0/22:6)	Phosphatidylcholine	PC Ester	HMDB07958	0.83	0.0224	0.1787
CE(22:5)	Cholesterol Ester	CE Ester	HMDB10375	0.85	0.0224	0.1787
PC(18:2/20:1)	Phosphatidylcholine	PC Ester	HMDB08144	0.82	0.0227	0.1787
PC(18:1/22:5)	Phosphatidylcholine	PC Ester	HMDB08122	0.86	0.0229	0.1787
PC(18:2/20:3)	Phosphatidylcholine	PC Ester	HMDB08146	0.79	0.0229	0.1787
PI(18:1/18:2)	Phosphatidylinositol	PI Ester	HMDB09838	0.83	0.0241	0.1852
PE(O-16:0/22:6)	Phosphatidylethanolamine	PE Ether		0.80	0.0245	0.1852
PI(18:0/20:4)	Phosphatidylinositol	PI Ester	HMDB09815	0.84	0.0246	0.1852
SM(24:0)	Sphingolipids	Sphingomyelin	HMDB11697	0.88	0.0256	0.1907

TAG53:6-FA18:3	Triacylglycerol	TAG Ester		0.63	0.0275	0.2028
PC(18:1/20:5)	Phosphatidylcholine	PC Ester	HMDB08116	0.78	0.0278	0.2031
CE(18:3)	Cholesterol Ester	CE Ester	HMDB10370	0.83	0.0283	0.2044
PE(18:1/20:1)	Phosphatidylethanolamine	PE Ester	HMDB09065	0.89	0.0287	0.2046
CE(20:3)	Cholesterol Ester	CE Ester	HMDB06736	0.83	0.0301	0.2123
CER(18:0)	Sphingolipids	Ceramide	HMDB04950	1.34	0.0305	0.2134
PE(P-18:1/20:4)	Phosphatidylethanolamine	PE Plasmalogen	HMDB11451	0.82	0.0314	0.2140
DCER(26:0)	Sphingolipids	Dihydroceramide	HMDB11771	0.85	0.0319	0.2140
LCER(14:0)	Sphingolipids	Lactosylceramide		0.79	0.0319	0.2140
PE(O-18:0/20:5)	Phosphatidylethanolamine	PE Ether		0.82	0.0327	0.2173
SM(20:1)	Sphingolipids	Sphingomyelin		0.87	0.0336	0.2208
CE(20:2)	Cholesterol Ester	CE Ester		0.85	0.0351	0.2232
PI(18:0/18:1)	Phosphatidylinositol	PI Ester		0.84	0.0353	0.2232
CER(24:0)	Sphingolipids	Ceramide	HMDB04956	0.84	0.0353	0.2232
TAG57:9-FA22:6	Triacylglycerol	TAG Ester		0.54	0.0353	0.2232
TAG50:6-FA20:4	Triacylglycerol	TAG Ester		0.72	0.0359	0.2232
PC(15:0/20:4)	Phosphatidylcholine	PC Ester	HMDB07949	0.86	0.0360	0.2232
TAG50:5-FA18:1	Triacylglycerol	TAG Ester		0.79	0.0375	0.2304
CE(20:5)	Cholesterol Ester	CE Ester	HMDB06731	0.81	0.0379	0.2306
PE(O-18:0/20:4)	Phosphatidylethanolamine	PE Ether		0.82	0.0400	0.2415
PC(14:0/22:6)	Phosphatidylcholine	PC Ester	HMDB07892	0.84	0.0417	0.2495
PC(18:2/22:5)	Phosphatidylcholine	PC Ester	HMDB08155	0.88	0.0425	0.2514
CE(20:4)	Cholesterol Ester	CE Ester	HMDB06726	0.86	0.0428	0.2514
PC(14:0/20:2)	Phosphatidylcholine	PC Ester	HMDB07880	0.94	0.0434	0.2528
PC(17:0/18:3)	Phosphatidylcholine	PC Ester		0.95	0.0458	0.2607
PC(14:0/18:2)	Phosphatidylcholine	PC Ester	HMDB07874	0.84	0.0459	0.2607
LPC(22:6)	Lysophosphatidylcholine	LPC Ester	HMDB10404	0.81	0.0462	0.2607
PC(18:1/20:4)	Phosphatidylcholine	PC Ester	HMDB08114	0.89	0.0465	0.2607
PE(O-16:0/22:5)	Phosphatidylethanolamine	PE Ether		0.81	0.0468	0.2607
PC(16:0/20:5)	Phosphatidylcholine	PC Ester	HMDB07984	0.81	0.0471	0.2607

Table S3. A summary of metabolites showing significantly different levels when comparing patients with and without early postconditional fluid overload. The table presents the name/identity of the metabolite along with the classification, the Human Metabolome Database (HMDB link), ratio, *p* value and *q* value for each individual metabolite. The ratio represents the median level for patients with versus patients without excessive fluid retention (green color indicates ratio <1.00, red color indicates ratio >1.00).

Biochemical name	Super pathway	Subpathway	HMDB	Ratio	*p* value	*q* value
PI(18:0/22:6)	Phosphatidylinositol	PI Ester	HMDB09821	0.79	0.0003	0.2342
PE(16:0/20:2)	Phosphatidylethanolamine	PE Ester	HMDB08934	0.93	0.0021	0.5350
PC(20:0/14:1)	Phosphatidylcholine	PC Ester	HMDB08263	0.78	0.0022	0.5350
TAG53:6-FA18:1	Triacylglycerol	TAG Ester		0.25	0.0046	0.6958
TAG53:5-FA18:1	Triacylglycerol	TAG Ester		0.66	0.0079	0.6958
PI(18:0/20:2)	Phosphatidylinositol	PI Ester	HMDB09812	0.88	0.0081	0.6958
PI(18:0/16:1)	Phosphatidylinositol	PI Ester	HMDB09806	0.78	0.0141	0.6958
PC(18:2/22:5)	Phosphatidylcholine	PC Ester	HMDB08155	0.85	0.0151	0.6958
LCER(22:1)	Sphingolipids	Lactosylceramide	HMDB11594	0.86	0.0167	0.6958
TAG55:7-FA20:3	Triacylglycerol	TAG Ester		0.43	0.0179	0.6958
CE(22:5)	Cholesterol Ester	CE Ester	HMDB10375	0.87	0.0181	0.6958
CE(22:6)	Cholesterol Ester	CE Ester	HMDB06733	0.82	0.0181	0.6958
PI(18:0/18:1)	Phosphatidylinositol	PI Ester		0.81	0.0190	0.6958
PE(18:1/20:1)	Phosphatidylethanolamine	PE Ester	HMDB09065	0.88	0.0207	0.6958
PC(20:0/22:6)	Phosphatidylcholine	PC Ester	HMDB08288	0.82	0.0237	0.6958
DCER(24:0)	Sphingolipids	Dihydroceramide	HMDB11768	0.86	0.0245	0.6958
PC(16:0/22:6)	Phosphatidylcholine	PC Ester	HMDB07991	0.83	0.0246	0.6958
LPE(16:1)	Lysophosphatidylethanolamine	LPE Ester	HMDB11474	0.79	0.0284	0.6958
PC(16:0/20:1)	Phosphatidylcholine	PC Ester	HMDB07978	0.87	0.0287	0.6958
PI(18:0/22:5)	Phosphatidylinositol	PI Ester	HMDB09820	0.81	0.0289	0.6958
TAG53:7-FA18:3	Triacylglycerol	TAG Ester		0.83	0.0310	0.6958
PI(16:0/16:1)	Phosphatidylinositol	PI Ester	HMDB09779	0.85	0.0340	0.6958
DAG(14:0/22:6)	Diacylglycerol	DAG Ester	HMDB07034	0.77	0.0345	0.6958
CE(16:1)	Cholesterol Ester	CE Ester	HMDB00658	0.83	0.0352	0.6958
TAG53:6-FA18:3	Triacylglycerol	TAG Ester		0.84	0.0354	0.6958
LCER(22:0)	Sphingolipids	Lactosylceramide	HMDB11594	0.89	0.0359	0.6958
PE(P-18:1/20:5)	Phosphatidylethanolamine	PE Plasmalogen	HMDB11420	0.75	0.0387	0.6958
PC(18:2/20:1)	Phosphatidylcholine	PC Ester	HMDB08144	0.85	0.0405	0.6958
CE(18:1)	Cholesterol Ester	CE Ester	HMDB00918	0.90	0.0433	0.6958
PC(16:0/22:5)	Phosphatidylcholine	PC Ester	HMDB07990	0.89	0.0441	0.6958
TAG58:6-FA16:0	Triacylglycerol	TAG Ester		0.83	0.0455	0.6958
LPE(20:1)	Lysophosphatidylethanolamine	LPE Ester	HMDB11482	0.83	0.0477	0.6958

cancers

Table S4. The metabolites showing statistically significant differences (Welch's two sample t-test, $p<0.05$) when comparing patients with versus without inflammation/fluid retention/acute GVHD. The table shows a summary of metabolites that are identified by at least two of these three comparisons. The table presents the identity of the metabolites along with the link to the Human Metabolome Database (HMDB), the relative ratio of the metabolite level for patients relative to the corresponding level for patients without inflammation/fluid retention/acute GVHD together with their *p* and *q* values. The green color indicates the relative ratios and shows that the levels were decreased for patients with inflammation/fluid retention/acute GVHD. Only two metabolites showed a similar significant difference for all three comparisons, and these metabolites are marked in yellow.

Biochemical Name	Super Pathway	Sub Pathway	Chemical ID	HMDB	RATIO With vs. without	RATIO With vs. without	Corresponding *p* and *r* values for the two comparisons			
							p value	*q* value	*p* value	*q* value
					INFLAMMATION	FLUID RETENTION				
PC(16:0/20:1)	Phosphatidylcholine	PC Ester	100009805	HMDB07978	0.83	0.87	0.0030	0.0774	0.0287	0.6958
PC(18:2/20:1)	Phosphatidylcholine	PC Ester	100009453	HMDB08144	0.82	0.85	0.0227	0.1787	0.0405	0.6958
PC(18:2/22:5)	Phosphatidylcholine	PC Ester	100009646	HMDB08155	0.88	0.85	0.0425	0.2514	0.0151	0.6958
PE(18:1/20:1)	Phosphatidylethanolamine	PE Ester	100009470	HMDB09065	0.89	0.88	0.0287	0.2046	0.0207	0.6958
PE(P-18:1/20:5)	Phosphatidylethanolamine	PE Plasmalogen	100010528	HMDB11420	0.68	0.75	0.0028	0.0768	0.0387	0.6958
PI(18:0/18:1)	Phosphatidylinositol	PI Ester	100009832		0.84	0.81	0.0353	0.2232	0.0190	0.6958
CE(18:1)	Cholesterol Ester	CE Ester	100010642	HMDB00918	0.87	0.90	0.0136	0.1413	0.0433	0.6958
CE(22:5)	Cholesterol Ester	CE Ester	100009945	HMDB10375	0.85	0.87	0.0224	0.1787	0.0181	0.6958
DCER(24:0)	Sphingolipids	Dihydroceramide	100010476	HMDB11768	0.83	0.86	0.0068	0.1075	0.0245	0.6958
LCER(22:0)	Sphingolipids	Lactosylceramide	100009788	HMDB11594	0.86	0.89	0.0086	0.1164	0.0359	0.6958
LCER(22:1)	Sphingolipids	Lactosylceramide	100010290	HMDB11594	0.85	0.86	0.0129	0.1358	0.0167	0.6958
TAG53:6-FA18:1	Triacylglycerol	TAG Ester	100010052		0.40	0.25	0.0005	0.0344	0.0046	0.6958
TAG53:6-FA18:3	Triacylglycerol	TAG Ester	100009714		0.63	0.84	0.0275	0.2028	0.0354	0.6958
TAG53:7-FA18:3	Triacylglycerol	TAG Ester	100010406		0.66	0.83	0.0172	0.1590	0.0310	0.6958
TAG55:7-FA20:3	Triacylglycerol	TAG Ester	100009724		0.43	0.43	0.0180	0.1594	0.0179	0.6958
					GVHD	FLUID RETENTION				
PC(16:0/20:1)	Phosphatidylcholine	PC Ester	100009805	HMDB07978	0.88	0.87	0.0461	0.1861	0.0287	0.6958

PC(18:2/22:5)	Phosphatidylcholine	PC Ester	100009646	HMDB08155	0.83	0.85	0.0031	0.1861	0.0151	0.6958
PC(20:0/14:1)	Phosphatidylcholine	PC Ester	100010498	HMDB08263	0.83	0.78	0.0371	0.1861	0.0022	0.5350
LPE(20:1)	Lysophosphatidylethanolamine	LPE Ester	100009795	HMDB11482	0.81	0.83	0.0263	0.1861	0.0477	0.6958
PI(18:0/20:2)	Phosphatidylinositol	PI Ester	100009676	HMDB09812	0.90	0.88	0.0468	0.1861	0.0081	0.6958
TAG58:6-FA16:0	Triacylglycerol	TAG Ester	100009737		0.69	0.83	0.0065	0.1861	0.0455	0.6958
					**GVHD**	**INFLAMMATION**				
PC(16:0/20:1)	Phosphatidylcholine	PC Ester	100009805	HMDB07978	0.88	0.83	0.0461	0.1861	0.0030	0.0774
PC(18:2/22:5)	Phosphatidylcholine	PC Ester	100009646	HMDB08155	0.83	0.88	0.0031	0.1861	0.0425	0.2514
PC(20:0/20:4)	Phosphatidylcholine	PC Ester	100009456	HMDB08279	0.79	0.75	0.0180	0.1861	0.0011	0.0496
DCER(26:0)	Sphingolipids	Dihydroceramide	100009782	HMDB11771	0.84	0.85	0.0257	0.1861	0.0319	0.2140
LCER(14:0)	Sphingolipids	Lactosylceramide	100009432		0.73	0.79	0.0054	0.1861	0.0319	0.2140
TAG57:9-FA22:6	Triacylglycerol	TAG Ester	100009561		0.13	0.54	0.0013	0.1861	0.0353	0.2232

cancers

Table S5. A summary of metabolites showing significantly different levels when comparing patients with and without steroid-requiring posttransplant acute GVHD. The table presents the name/identity of metabolites along with the classification, the Human Metabolome Database (HMDB link), ratio, *p* value and *q* value for each individual metabolite. The ratio represents the median level for patients with versus patients without acute GVHD (green color indicates ratio <1.00, red color indicates ratio >1.00).

Biochemical name	Super pathway	Subpathway	HMDB	Ratio	*p* value	*q* value
TAG57:9-FA22:6	Triacylglycerol	TAG Ester		0.13	0.0013	0.1861
PC(18:2/22:5)	Phosphatidylcholine	PC Ester	HMDB08155	0.83	0.0031	0.1861
LCER(14:0)	Sphingolipids	Lactosylceramide		0.73	0.0054	0.1861
TAG58:6-FA16:0	Triacylglycerol	TAG Ester		0.69	0.0065	0.1861
DAG(18:1/20:2)	Diacylglycerol	DAG Ester	HMDB07225	0.72	0.0075	0.1861
TAG58:7-FA16:0	Triacylglycerol	TAG Ester		0.72	0.0094	0.1861
DAG(18:1/22:5)	Diacylglycerol	DAG Ester	HMDB07236	0.69	0.0104	0.1861
MAG(15:0)	Monoacylgycerol	MAG Ester		0.52	0.0116	0.1861
MAG(20:1)	Monoacylgycerol	MAG Ester		0.55	0.0120	0.1861
DAG(16:0/20:5)	Diacylglycerol	DAG Ester	HMDB07114	0.63	0.0128	0.1861
LCER(16:0)	Sphingolipids	Lactosylceramide	HMDB06750	0.77	0.0132	0.1861
MAG(22:4)	Monoacylgycerol	MAG Ester		0.51	0.0141	0.1861
DAG(16:0/22:5)	Diacylglycerol	DAG Ester	HMDB07120	0.72	0.0145	0.1861
DAG(18:1/20:1)	Diacylglycerol	DAG Ester	HMDB07224	0.77	0.0146	0.1861
MAG(18:1)	Monoacylgycerol	MAG Ester		0.38	0.0157	0.1861
Total MAG	Neutral Complex Lipids SUM	Monoacylglycerols		0.40	0.0158	0.1861
MAG(22:5)	Monoacylgycerol	MAG Ester		0.44	0.0164	0.1861
TAG54:3-FA16:1	Triacylglycerol	TAG Ester		0.73	0.0166	0.1861
MAG(16:1)	Monoacylgycerol	MAG Ester		0.31	0.0173	0.1861
DAG(18:0/22:6)	Diacylglycerol	DAG Ester	HMDB07179	0.70	0.0177	0.1861
PC(20:0/20:4)	Phosphatidylcholine	PC Ester	HMDB08279	0.79	0.0180	0.1861
DAG(18:2/22:5)	Diacylglycerol	DAG Ester	HMDB07265	0.69	0.0180	0.1861
DAG(16:0/20:3)	Diacylglycerol	DAG Ester	HMDB07111	0.71	0.0182	0.1861
DAG(16:1/20:2)	Diacylglycerol	DAG Ester	HMDB07138	0.79	0.0183	0.1861
MAG(17:0)	Monoacylgycerol	MAG Ester		0.65	0.0187	0.1861
MAG(18:2)	Monoacylgycerol	MAG Ester		0.44	0.0193	0.1861
DAG(18:1/20:3)	Diacylglycerol	DAG Ester	HMDB07227	0.71	0.0195	0.1861
MAG(22:6)	Monoacylgycerol	MAG Ester		0.67	0.0198	0.1861
DAG(18:2/20:3)	Diacylglycerol	DAG Ester	HMDB07256	0.71	0.0206	0.1861
DAG(16:1/22:6)	Diacylglycerol	DAG Ester	HMDB07150	0.67	0.0207	0.1861
DAG(14:1/16:0)	Diacylglycerol	DAG Ester	HMDB07096	0.70	0.0211	0.1861
MAG(18:3)	Monoacylgycerol	MAG Ester		0.55	0.0218	0.1861
DAG(16:0/16:0)	Diacylglycerol	DAG Ester	HMDB07098	0.52	0.0232	0.1861
TAG54:4-FA16:1	Triacylglycerol	TAG Ester		0.78	0.0234	0.1861
DAG(18:2/20:5)	Diacylglycerol	DAG Ester	HMDB07259	0.61	0.0255	0.1861
DCER(26:0)	Sphingolipids	Dihydroceramide	HMDB11771	0.84	0.0257	0.1861
DAG(18:1/20:5)	Diacylglycerol	DAG Ester	HMDB07230	0.67	0.0257	0.1861
DAG(18:1/22:6)	Diacylglycerol	DAG Ester	HMDB07237	0.63	0.0261	0.1861
LPE(20:1)	Lysophosphatidylethanolamine	LPE Ester	HMDB11482	0.81	0.0263	0.1861
DAG(18:2/22:4)	Diacylglycerol	DAG Ester	HMDB07263	0.77	0.0264	0.1861

DAG(16:0/22:6)	Diacylglycerol	DAG Ester	HMDB07121	0.67	0.0274	0.1861
MAG(12:0)	Monoacylgycerol	MAG Ester		0.52	0.0274	0.1861
DAG(18:1/22:4)	Diacylglycerol	DAG Ester	HMDB07234	0.77	0.0276	0.1861
DAG(14:0/16:0)	Diacylglycerol	DAG Ester	HMDB07095	0.61	0.0280	0.1861
PE(P-18:2/18:2)	Phosphatidylethanolamine	PE Plasmalogen	HMDB09093	0.88	0.0282	0.1861
DAG(14:0/16:1)	Diacylglycerol	DAG Ester	HMDB07012	0.66	0.0283	0.1861
MAG(20:2)	Monoacylgycerol	MAG Ester		0.47	0.0304	0.1861
MAG(14:1)	Monoacylgycerol	MAG Ester		0.41	0.0307	0.1861
DAG(16:1/18:2)	Diacylglycerol	DAG Ester	HMDB07132	0.72	0.0310	0.1861
TAG58:5-FA18:1	Triacylglycerol	TAG Ester		0.80	0.0310	0.1861
Total DAG	Neutral Complex Lipids SUM	Diacylglycerols		0.76	0.0335	0.1861
DAG(16:1/16:1)	Diacylglycerol	DAG Ester	HMDB07128	0.63	0.0338	0.1861
DAG(18:2/22:6)	Diacylglycerol	DAG Ester	HMDB07266	0.62	0.0347	0.1861
MAG(20:3)	Monoacylgycerol	MAG Ester		0.47	0.0358	0.1861
TAG54:7-FA22:5	Triacylglycerol	TAG Ester		0.74	0.0365	0.1861
MAG(14:0)	Monoacylgycerol	MAG Ester		0.28	0.0369	0.1861
PC(20:0/14:1)	Phosphatidylcholine	PC Ester	HMDB08263	0.83	0.0371	0.1861
TAG56:4-FA20:2	Triacylglycerol	TAG Ester		0.80	0.0384	0.1861
DAG(16:1/18:0)	Diacylglycerol	DAG Ester	HMDB07129	0.77	0.0396	0.1861
DAG(16:0/16:1)	Diacylglycerol	DAG Ester	HMDB07099	0.65	0.0402	0.1861
TAG58:7-FA22:5	Triacylglycerol	TAG Ester		0.78	0.0414	0.1861
MAG(20:5)	Monoacylgycerol	MAG Ester		0.76	0.0420	0.1861
HCER(18:0)	Sphingolipids	Hexosylceramide	HMDB04972	1.21	0.0428	0.1861
TAG56:3-FA16:0	Triacylglycerol	TAG Ester		0.78	0.0430	0.1861
TAG56:7-FA16:1	Triacylglycerol	TAG Ester		0.78	0.0441	0.1861
PC(18:2/20:2)	Phosphatidylcholine	PC Ester	HMDB08145	0.82	0.0442	0.1861
TAG56:8-FA16:1	Triacylglycerol	TAG Ester		0.71	0.0447	0.1861
DAG(18:0/18:3)	Diacylglycerol	DAG Ester	HMDB07163	0.82	0.0460	0.1861
PC(16:0/20:1)	Phosphatidylcholine	PC Ester	HMDB07978	0.88	0.0461	0.1861
PI(18:0/20:2)	Phosphatidylinositol	PI Ester	HMDB09812	0.90	0.0468	0.1861
TAG58:7-FA18:1	Triacylglycerol	TAG Ester		0.79	0.0475	0.1861

cancers

Table S6. The classification of lipid metabolites showing significantly different levels when comparing patients with and without pretransplant inflammation, early postconditioning fluid overload and posttransplant acute GVHD. The table shows the number of different lipid metabolites (and their percentages) in the six subclusters identified in the unsupervised hirerarchical cluster analysis, based on the lipid metabolites that differed significantly between patients with and without inflammation/fluid overload/acute GVHD (see Figure S2).

**Metabolite main class**	**Metabolite subclass**	**Subcluster**			**Subcluster**		
		**1.1**	**1.2**	**1.3**	**2.1**	**2.2**	**2.3**
**Number of metabolites in cluster**		**38**	**41**	**24**	**22**	**24**	**20**
Phosphatidylcholine	PC Ester	9 (24%)		3 (13%)	8 (36%)	6 (25%)	
Lysophosphatidylcholine	LPC Ester				1 (5%)	2 (8%)	14 (70%)
Phosphatidylethanolamine	PE Ester/Ether/ Plasmalogen	11 (29%)			6 (27%)	10 (42%)	
Lysophosphatidylethanolamine	LPE Ester			1 (4%)			2 (10%)
Phosphatidylinositol	PI Ester			2 (8%)		3 (13%)	
Cholesteryl Ester	CE Ester	12 (32%)		1 (4%)	3 (14%)		
Sphingolipids	Ceramide	2 (5%)		1 (4%)			
	Dihydroceramide	1 (3%)		1 (4%)			
	Hexosylceramide			1 (4%)			
	Lactosylceramide				2 (9%)		4 (20%)
	Sphingomyelin	3 (8%)					
Monoacylglycerol	MAG Ester			17 (71%)			
Diacylglycerol	DAG Ester		27 (66%)	1 (4%)	1 (5%)		
Triacylglycerol	TAG Ester		14 (34%)		1 (5%)	3 (13%)	

Table S7. The differences in serum lipid profiles between allotransplant recipients with and without preconditioning signs of inflammation; a summary of important biological characteristics for lipid biochemical subclasses investigated in the present study. The table is based on information given in the Gene database, the Human Metabolome Database and selected references from the PubMed database.

**Classification (main group and subgroup)**	**Comments**
Phosphatidylcholine<br>PC ester	Phosphatidylcholine comprises 40-50% of total cellular phospholipids and it is synthesized by all mammalian cells. It is also important for the lipoprotein release by liver cells. At least for some cells the biological effect depends on the phosphatidylcholine:phosphatidylethanolamine molar ratio. Phosphatidylcholine is important for gastrointestinal lipid uptake and also for formation of the protective hydrophobic mucus layer. Metabolism of the sulfur amino acids methionine and cysteine will influence phosphatidylcholine metabolism [2,3].
Lysophosphatidylcholine<br>LPC ester	Lysophospholipids are deacetylated forms of phospholipids, and lysophosphosphatidylcholine is the most abundant subclass. They serve as precursors for diacyl phospholipid mediators. Lysophosphatodylcholines are ligands for TLR2 and TLR4 and possibly also for the G2A and GPR4 receptors. G2A has been identified as a G protein-coupled receptor that can be induced by different classes of DNA-damaging agents and block cell cycle progression in lymphocytes. It is reported that G2A functions as a receptor for oxidized free fatty acids derived from linoleic and arachidonic acids [4]. GPR4 can bind both sphingosylphosphorylcholine and lysophosphatidylcholine and is thereby a regulator of vascular permeability and also inflammation, including gastrointestinal inflammation [5-7].These lipids stimulate the release of proinflammatory cytokines, inhibits migration and proliferation of endothelial cells, activates and polarizes macrophages towards the M1 phenotype, activates B cells and induces Treg differentiation [8-10].
Phosphatidylethanolamine<br>PE ester<br>Phosphatidylethanolamine<br>PE plasmalogen	Phosphatidylethanolamines are the second most abundant phospholipids in mammalian membranes (15-25 % of total lipids); they are synthesized from ethanolamine and are enriched in the inner membrane. Mitochondria are rich in phosphatidyletanolamines. It is also important for the lipoprotein release by the liver cells. At least for some cells, the biological effect depends on the phosphatidylcholine:phosphatidylethanolamine molar ratio.<br>Phosphatidylethanolamines functions as lipid chaperones and is also important for the synthesis of glycosylphosphatidylinositol-anchored proteins. Other functions are stimulation of OXPHOS, regulation of autophagy and regulation of ferroptotic cell death [2,11].
Lysophosphatidylethanolam ine<br>LPE ester	Experimental evidence suggests that (some of) these biochemicals have mediator functions, but the effects and the possible receptor ligation has not been thoroughly investigated. However, a recent study suggests that such lipids can stimulate growth through ligation of G-protein coupled receptors [10,12,13].
Phosphatidylinositol<br>PI ester	These are structural phospholipids that serve as membrane-anchored precursors of the regulatory polyphosphoinositide lipids; these lipids constitute a minor part of the plasma membrane but are central regulators of cellular physiology and intracellular signaling. The polyphosphoinositides interact with proteins both through nonspecific electrostatic forces and specific recognition, and this protein recruitment to the plasma membrane is further orchestrated through their phosphorylation status [14,15].
Cholesteryl Ester	These are formed by the esterification of cholesterol with long-chain fatty acids and contribute to transport of cholesterols through the blood by lipoproteins. Increased levels of intracellular cholesteryl esters indicate abnormal cholesterol metabolism.

	Cholesterol is an essential structural component of cell membranes. However, cholesterol also regulates the biological functions through molecular interactions with sphingomyelins and possibly formation of ceramide-cholesterol complexes. Cholesterol can also function as a regulator of cell cycle progression, especially G_1–S transition, and this effect is associated with inhibition of ERK1/2 signaling and reduced cyclin D1 [16,17].
Sphingolipid Ceramide<br>Sphingolipid Dihydroceramide<br>Sphingolipid Hexosylceramide<br><br>Sphingolipid Lactosylceramide	Ceramides are bioactive lipids that are regulators of many biological processes both at the cellular level (e.g., proapoptotic effects through interactions with both p53 and CD95) and inflammation. They are synthesized in the cells and function as a coordinator of stress responses; increased synthesis can be caused by chemotherapy, environmental stress and proinflammatory cytokines. Scoring systems have been suggested to evaluate the clinical impact of the systemic levels of various ceramides. Ceramides thus have an opposite effect to sphingosine-1-phosphate [18-21]. There is a close interaction between ceramide and sphingosine synthesis; including sphingosine-1-phosphate that serves as a mediator, binds specific receptors (S1PR1-5) and is an important regulator of both T cell activation and endothelial functions [22-26]. Previous studies suggest that systemic ceramide levels can be markers of both cardiovascular dysfunction and cancer death [27,28].<br><br>Ceramide seems to be important for chemosensitivity in human AML [29,30].
Sphingolipid Sphingomyelin	Sphingomyelin is a component of the external leaflet of the plasma membrane (also enriched in endosomes and Golgi network); it interacts with cholesterol and this interaction modulates its biological functions. Sphingomyelin-cholesterol is important for recruitment of (plasma) membrane proteins. Finally, sphingomyelin is a precursor to ceramide [31,32].
Diacylglycerol DAG ester<br><br>Triacylglycerol TAG ester<br><br>Monoacylglycerol MAG Ester	Diacylglycerols are precursors of triacylglycerols; they are also common food additives [33-35].<br>Triacylglycerols or triglycerides are the main storage molecules of metabolic energy [33-35].<br>Monoacylglycerols are formed by lipolysis. The monoacylglycerol lipase hydrolyses monoglycerides into glycerol and various fatty acids with different length and saturation in this last step of triacylglycerol catabolism. This general enzymatic activity will thereby also influence regulation of inflammation through its effects on 2-arachidonoyl glycerol and the levels of ligands for cannabinoid receptors and the synthesis of prostaglandins [36].<br><br>There are functional/synthetic interactions between fatty acid synthesis, fatty acid uptake and sphingolipid metabolism [37-39]. Ceramides are involved in these interactions that are important for the regulation of inflammation and the function of monocytes [40-43].

References

1. Lofgren, L.; Stahlman, M.; Forsberg, G.B.; Saarinen, S.; Nilsson, R.; Hansson, G.I. The BUME method: a novel automated chloroform-free 96-well total lipid extraction method for blood plasma. *J Lipid Res* **2012**, *53*, 1690-1700, doi:10.1194/jlr.D023036.
2. van der Veen, J.N.; Kennelly, J.P.; Wan, S.; Vance, J.E.; Vance, D.E.; Jacobs, R.L. The critical role of phosphatidylcholine and phosphatidylethanolamine metabolism in health and disease. *Biochim Biophys Acta Biomembr* **2017**, *1859*, 1558-1572, doi:10.1016/j.bbamem.2017.04.006.
3. Blachier, F.; Andriamihaja, M.; Blais, A. Sulfur-Containing Amino Acids and Lipid Metabolism. *J Nutr* **2020**, *150*, 2524S-2531S, doi:10.1093/jn/nxaa243.
4. Obinata, H.; Izumi, T. G2A as a receptor for oxidized free fatty acids. *Prostaglandins Other Lipid Mediat* **2009**, *89*, 66-72, doi:10.1016/j.prostaglandins.2008.11.002.

5. Kim, K.S.; Ren, J.; Jiang, Y.; Ebrahem, Q.; Tipps, R.; Cristina, K.; Xiao, Y.J.; Qiao, J.; Taylor, K.L.; Lum, H., et al. GPR4 plays a critical role in endothelial cell function and mediates the effects of sphingosylphosphorylcholine. *FASEB J* **2005**, *19*, 819-821, doi:10.1096/fj.04-2988fje.
6. Qiao, J.; Huang, F.; Naikawadi, R.P.; Kim, K.S.; Said, T.; Lum, H. Lysophosphatidylcholine impairs endothelial barrier function through the G protein-coupled receptor GPR4. *Am J Physiol Lung Cell Mol Physiol* **2006**, *291*, L91-101, doi:10.1152/ajplung.00508.2005.
7. Wang, Y.; de Valliere, C.; Imenez Silva, P.H.; Leonardi, I.; Gruber, S.; Gerstgrasser, A.; Melhem, H.; Weber, A.; Leucht, K.; Wolfram, L., et al. The Proton-activated Receptor GPR4 Modulates Intestinal Inflammation. *J Crohns Colitis* **2018**, *12*, 355-368, doi:10.1093/ecco-jcc/jjx147.
8. Law, S.H.; Chan, M.L.; Marathe, G.K.; Parveen, F.; Chen, C.H.; Ke, L.Y. An Updated Review of Lysophosphatidylcholine Metabolism in Human Diseases. *Int J Mol Sci* **2019**, *20*, doi:10.3390/ijms20051149.
9. Liu, P.; Zhu, W.; Chen, C.; Yan, B.; Zhu, L.; Chen, X.; Peng, C. The mechanisms of lysophosphatidylcholine in the development of diseases. *Life Sci* **2020**, *247*, 117443, doi:10.1016/j.lfs.2020.117443.
10. Makide, K.; Uwamizu, A.; Shinjo, Y.; Ishiguro, J.; Okutani, M.; Inoue, A.; Aoki, J. Novel lysophosphoplipid receptors: their structure and function. *J Lipid Res* **2014**, *55*, 1986-1995, doi:10.1194/jlr.R046920.
11. Patel, D.; Witt, S.N. Ethanolamine and Phosphatidylethanolamine: Partners in Health and Disease. *Oxid Med Cell Longev* **2017**, *2017*, 4829180, doi:10.1155/2017/4829180.
12. Hisano, K.; Kawase, S.; Mimura, T.; Yoshida, H.; Yamada, H.; Haniu, H.; Tsukahara, T.; Kurihara, T.; Matsuda, Y.; Saito, N., et al. Structurally different lysophosphatidylethanolamine species stimulate neurite outgrowth in cultured cortical neurons via distinct G-protein-coupled receptors and signaling cascades. *Biochem Biophys Res Commun* **2021**, *534*, 179-185, doi:10.1016/j.bbrc.2020.11.119.
13. Makide, K.; Kitamura, H.; Sato, Y.; Okutani, M.; Aoki, J. Emerging lysophospholipid mediators, lysophosphatidylserine, lysophosphatidylthreonine, lysophosphatidylethanolamine and lysophosphatidylglycerol. *Prostaglandins Other Lipid Mediat* **2009**, *89*, 135-139, doi:10.1016/j.prostaglandins.2009.04.009.
14. Pemberton, J.G.; Balla, T. Polyphosphoinositide-Binding Domains: Insights from Peripheral Membrane and Lipid-Transfer Proteins. *Adv Exp Med Biol* **2019**, *1111*, 77-137, doi:10.1007/5584_2018_288.
15. Pemberton, J.G.; Kim, Y.J.; Balla, T. Integrated regulation of the phosphatidylinositol cycle and phosphoinositide-driven lipid transport at ER-PM contact sites. *Traffic* **2020**, *21*, 200-219, doi:10.1111/tra.12709.
16. Batetta, B.; Mulas, M.F.; Sanna, F.; Putzolu, M.; Bonatesta, R.R.; Gasperi-Campani, A.; Roncuzzi, L.; Baiocchi, D.; Dessi, S. Role of cholesterol ester pathway in the control of cell cycle in human aortic smooth muscle cells. *FASEB J* **2003**, *17*, 746-748, doi:10.1096/fj.02-0396fje.
17. Garcia-Arribas, A.B.; Alonso, A.; Goni, F.M. Cholesterol interactions with ceramide and sphingomyelin. *Chem Phys Lipids* **2016**, *199*, 26-34, doi:10.1016/j.chemphyslip.2016.04.002.
18. Ghosh, S.; Juin, S.K.; Majumdar, S. Cancer stem cells and ceramide signaling: the cutting edges of immunotherapy. *Mol Biol Rep* **2020**, *47*, 8101-8111, doi:10.1007/s11033-020-05790-z.
19. Hilvo, M.; Vasile, V.C.; Donato, L.J.; Hurme, R.; Laaksonen, R. Ceramides and Ceramide Scores: Clinical Applications for Cardiometabolic Risk Stratification. *Front Endocrinol (Lausanne)* **2020**, *11*, 570628, doi:10.3389/fendo.2020.570628.
20. Jeffries, K.A.; Krupenko, N.I. Ceramide Signaling and p53 Pathways. *Adv Cancer Res* **2018**, *140*, 191-215, doi:10.1016/bs.acr.2018.04.011.
21. Moro, K.; Nagahashi, M.; Gabriel, E.; Takabe, K.; Wakai, T. Clinical application of ceramide in cancer treatment. *Breast Cancer* **2019**, *26*, 407-415, doi:10.1007/s12282-019-00953-8.
22. Cartier, A.; Hla, T. Sphingosine 1-phosphate: Lipid signaling in pathology and therapy. *Science* **2019**, *366*, doi:10.1126/science.aar5551.
23. Hait, N.C.; Maiti, A. The Role of Sphingosine-1-Phosphate and Ceramide-1-Phosphate in Inflammation and Cancer. *Mediators Inflamm* **2017**, *2017*, 4806541, doi:10.1155/2017/4806541.
24. Gault, C.R.; Obeid, L.M.; Hannun, Y.A. An overview of sphingolipid metabolism: from synthesis to breakdown. *Adv Exp Med Biol* **2010**, *688*, 1-23, doi:10.1007/978-1-4419-6741-1_1.
25. O'Sullivan, C.; Dev, K.K. The structure and function of the S1P1 receptor. *Trends Pharmacol Sci* **2013**, *34*, 401-412, doi:10.1016/j.tips.2013.05.002.
26. Xiong, Y.; Hla, T. S1P control of endothelial integrity. *Curr Top Microbiol Immunol* **2014**, *378*, 85-105, doi:10.1007/978-3-319-05879-5_4.
27. Petrocelli, J.J.; McKenzie, A.I.; Mahmassani, Z.S.; Reidy, P.T.; Stoddard, G.J.; Poss, A.M.; Holland, W.L.; Summers, S.A.; Drummond, M.J. Ceramide Biomarkers Predictive of Cardiovascular Disease Risk Increase in Healthy Older Adults After Bed Rest. *J Gerontol A Biol Sci Med Sci* **2020**, *75*, 1663-1670, doi:10.1093/gerona/glaa072.
28. Walker, M.E.; Xanthakis, V.; Peterson, L.R.; Duncan, M.S.; Lee, J.; Ma, J.; Bigornia, S.; Moore, L.L.; Quatromoni, P.A.; Vasan, R.S., et al. Dietary Patterns, Ceramide Ratios, and Risk of All-Cause and Cause-Specific Mortality: The Framingham Offspring Study. *J Nutr* **2020**, *150*, 2994-3004, doi:10.1093/jn/nxaa269.

29. Pallis, M.; Russell, N. P-glycoprotein plays a drug-efflux-independent role in augmenting cell survival in acute myeloblastic leukemia and is associated with modulation of a sphingomyelin-ceramide apoptotic pathway. *Blood* **2000**, *95*, 2897-2904.
30. Plo, I.; Bettaieb, A.; Payrastre, B.; Mansat-De Mas, V.; Bordier, C.; Rousse, A.; Kowalski-Chauvel, A.; Laurent, G.; Lautier, D. The phosphoinositide 3-kinase/Akt pathway is activated by daunorubicin in human acute myeloid leukemia cell lines. *FEBS Lett* **1999**, *452*, 150-154, doi:10.1016/s0014-5793(99)00631-6.
31. Ramstedt, B.; Slotte, J.P. Membrane properties of sphingomyelins. *FEBS Lett* **2002**, *531*, 33-37, doi:10.1016/s0014-5793(02)03406-3.
32. Slotte, J.P. Biological functions of sphingomyelins. *Prog Lipid Res* **2013**, *52*, 424-437, doi:10.1016/j.plipres.2013.05.001.
33. Bhatt-Wessel, B.; Jordan, T.W.; Miller, J.H.; Peng, L. Role of DGAT enzymes in triacylglycerol metabolism. *Arch Biochem Biophys* **2018**, *655*, 1-11, doi:10.1016/j.abb.2018.08.001.
34. Quiroga, A.D.; Lehner, R. Pharmacological intervention of liver triacylglycerol lipolysis: The good, the bad and the ugly. *Biochem Pharmacol* **2018**, *155*, 233-241, doi:10.1016/j.bcp.2018.07.005.
35. Yen, C.L.; Stone, S.J.; Koliwad, S.; Harris, C.; Farese, R.V., Jr. Thematic review series: glycerolipids. DGAT enzymes and triacylglycerol biosynthesis. *J Lipid Res* **2008**, *49*, 2283-2301, doi:10.1194/jlr.R800018-JLR200.
36. Deng, H.; Li, W. Monoacylglycerol lipase inhibitors: modulators for lipid metabolism in cancer malignancy, neurological and metabolic disorders. *Acta Pharm Sin B* **2020**, *10*, 582-602, doi:10.1016/j.apsb.2019.10.006.
37. Bottolo, L.; Miller, S.; Johnson, S.R. Sphingolipid, fatty acid and phospholipid metabolites are associated with disease severity and mTOR inhibition in lymphangioleiomyomatosis. *Thorax* **2020**, *75*, 679-688, doi:10.1136/thoraxjnl-2019-214241.
38. Jafari, N.; Drury, J.; Morris, A.J.; Onono, F.O.; Stevens, P.D.; Gao, T.; Liu, J.; Wang, C.; Lee, E.Y.; Weiss, H.L., et al. De Novo Fatty Acid Synthesis-Driven Sphingolipid Metabolism Promotes Metastatic Potential of Colorectal Cancer. *Mol Cancer Res* **2019**, *17*, 140-152, doi:10.1158/1541-7786.MCR-18-0199.
39. Park, W.J.; Park, J.W.; Merrill, A.H.; Storch, J.; Pewzner-Jung, Y.; Futerman, A.H. Hepatic fatty acid uptake is regulated by the sphingolipid acyl chain length. *Biochim Biophys Acta* **2014**, *1841*, 1754-1766, doi:10.1016/j.bbalip.2014.09.009.
40. Kucuk, S.; Niven, J.; Caamano, J.; Jones, S.W.; Camacho-Munoz, D.; Nicolaou, A.; Mauro, C. Unwrapping the mechanisms of ceramide and fatty acid-initiated signals leading to immune-inflammatory responses in obesity. *Int J Biochem Cell Biol* **2021**, *135*, 105972, doi:10.1016/j.biocel.2021.105972.
41. Fucho, R.; Casals, N.; Serra, D.; Herrero, L. Ceramides and mitochondrial fatty acid oxidation in obesity. *FASEB J* **2017**, *31*, 1263-1272, doi:10.1096/fj.201601156R.
42. Nishiyama, K.; Fujita, T.; Fujimoto, Y.; Nakajima, H.; Takeuchi, T.; Azuma, Y.T. Fatty acid transport protein 1 enhances the macrophage inflammatory response by coupling with ceramide and c-Jun N-terminal kinase signaling. *Int Immunopharmacol* **2018**, *55*, 205-215, doi:10.1016/j.intimp.2017.12.003.
43. Zhang, Y.; Hao, J.; Sun, Y.; Li, B. Saturated Fatty Acids Induce Ceramide-associated Macrophage Cell Death. *J Vis Exp* **2017**, 10.3791/56535, doi:10.3791/56535.
